# Efficient EM Estimation for the Pogit Model via Polya-Gamma Augmentation

**DOI:** 10.3390/e28020207

**Published:** 2026-02-11

**Authors:** Iván Gutiérrez, Sandra Ramírez, Leonardo Jofré

**Affiliations:** 1 Departamento de Economía y Administración, Facultad de Economía y Negocios, Universidad Andrés Bello, Santiago 8370134, Chile; 2 Departamento de Ciencias Naturales y Matemáticas, Facultad de Ingeniería y Ciencias, Pontificia Universidad Javeriana, Cali 760031, Colombia; 3 Departamento de Estadística, Facultad de Matemáticas, Pontificia Universidad Católica de Chile, Santiago 7820436, Chile; lnjofre@mat.uc.cl

**Keywords:** EM algorithm, pogit, under-reporting, share-of-wallet

## Abstract

The Poisson-logistic (pogit) model is widely used for count data with latent intensities, with applications including under-reporting correction and share-of-wallet estimation, yet existing estimation methods do not scale well to large datasets. We propose a new expectation-maximization (EM) algorithm for the standard pogit model based on Polya-Gamma data augmentation, which yields a conditionally Gaussian complete-data likelihood with closed-form EM-updates. The resulting EM algorithm has low per-iteration cost and naturally accommodates computational enhancements, including quasi-Newton acceleration and mini-batch implementations. These features enable efficient inference on datasets with millions of observations. Simulation studies and real-data applications demonstrate substantial computational improvements without loss of statistical accuracy, and comparisons with direct maximum-likelihood optimization routines show that the proposed method provides a scalable and competitive alternative for large-scale pogit estimation.

## 1. Introduction

Count data models (see, e.g., [1]) with latent exposure or reporting mechanisms are common in many empirical settings, including marketing analytics [2], epidemiology [3], official statistics [4], and gender-violence research [5,6]. Among these, the Poisson-logistic (pogit) model [7] has emerged as a flexible and interpretable framework for modeling observed counts subject to under-reporting or partial observability. By combining a binomial observation equation with a Poisson exposure model, the pogit specification allows researchers to disentangle reporting behavior from the underlying intensity process, while retaining a regression structure that facilitates inference and interpretation.

The pogit model has found applications in diverse areas. In studies of under-reporting, it provides a principled way to account for missing or censored events by explicitly modeling the probability that an event is observed (see, e.g., [7]). In marketing and consumer analytics, it has been used for share-of-wallet (SoW) estimation, where observed purchases represent a noisy subset of latent purchase opportunities (see, e.g., [2]). These applications have motivated a growing methodological literature, including extensions to negative-binomial exposures [4], as well as parametric and nonparametric Bayesian formulations that incorporate prior information and/or variable selection (see, e.g., [5,8,9]).

Despite these advances, a major practical limitation of existing pogit methodologies is their lack of scalability. While the latent count can be analytically marginalized and maximum-likelihood estimation can in principle be based on the observed data likelihood, the resulting objective function is highly nonlinear and tightly couples the reporting and intensity components through a multiplicative structure. This makes direct likelihood maximization numerically delicate in practice, particularly as sample size and covariate dimension grow, or when parameters are weakly identified. Bayesian approaches face related difficulties, as Markov chain Monte Carlo methods must explore high–dimensional posteriors with strong dependence across model components. As a consequence, pogit models remain difficult to deploy in large–scale applications, despite their conceptual suitability for precisely such settings.

In this article, we address this literature gap by introducing a new expectation-maximization (EM) algorithm [10] for efficient estimation of the standard pogit model. Our approach builds on Polya-Gamma data augmentation [11], which yields conditionally Gaussian complete-data likelihoods and enables closed-form updates in the M-step. Specifically, we adapt the Polya-Gamma-based EM framework developed by Scott et al. [12] for logistic regression to the pogit setting, combining it with the approximate augmentation strategy for Poisson models introduced by D’Angelo et al. [13]. This synthesis results in an EM algorithm with a simple structure and closed–form updates throughout. Crucially, the resulting procedure is naturally amenable to online and mini-batch variants, making it possible to fit pogit models to datasets of unprecedented size using streaming data, without sacrificing statistical efficiency.

The remainder of the article is organized as follows. Section 2 introduces the pogit model and reviews its likelihood structure. Section 3 presents the proposed Polya-Gamma-based EM algorithm and discusses its computational properties, including quasi-Newton acceleration and mini-batch extensions for large-scale data. Section 4 describes additional computational enhancements. Section 5 reports results from simulation studies assessing convergence, finite-sample performance, and computational efficiency, with comparisons to direct numerical maximum-likelihood estimation. Section 6 applies the method to real datasets. Section 7 concludes.

### Contributions

This article makes the following contributions:

- We introduce a new expectation–maximization algorithm for the standard pogit model based on Polya-Gamma data augmentation. By combining an exact augmentation for the binomial component with a controlled approximation for the Poisson component, the complete–data log–likelihood becomes quadratic in the regression parameters. This yields closed-form expressions for all E-step expectations and reduces the M-step to simple weighted least-squares updates, resulting in a fully analytic EM procedure with low per-iteration computational cost.
- We show that the resulting EM algorithm admits scalable online and mini-batch variants. In particular, the method can be applied to datasets with millions of observations using mini-batch updates, making pogit models feasible in large-scale applications where existing methods break down.
- We evaluate the statistical and computational performance of the proposed estimator using both simulated and real datasets. The results demonstrate fast convergence, stable behavior across sample sizes, and competitive estimation accuracy.
- We provide a systematic comparison with direct numerical maximization of the observed-data likelihood using generic maximum-likelihood routines, showing that the proposed method delivers substantial runtime improvements while exhibiting stable finite-sample behavior, robust parameter recovery, and numerical stability.

## 2. Model

### 2.1. Hierarchical Specification

We consider the standard Poisson-logistic (pogit) model for count data with latent intensity and partial observability. For each observational unit i=1,…,N, let $y_{i}$ denote the observed count and $n_{i}$ an unobserved latent count representing the total number of underlying events or opportunities. The model is defined hierarchically as

$$\begin{array}{c} \left(y_{i}|n_{i},\theta_{i}\right)\overset{ind}{∼}\mathrm{Bin}\left(n_{i},\theta_{i}\right),\\ \left(n_{i}|\lambda_{i}\right)\overset{ind}{∼}\mathrm{Poisson}\left(E_{i}\lambda_{i}\right), \end{array}\;i=1,\ldots ,N,$$

where $\overset{ind}{∼}$ denotes conditional independence across observational units, conditional on the model parameters and covariates, $E_{i}$ is a known offset, $\theta_{i}\in \left(0,1\right)$ is the probability that a latent event is observed, and $\lambda_{i}>0$ is the latent intensity. The first-level binomial equation captures partial observability: conditional on $n_{i}$, only a fraction $\theta_{i}$ of events is recorded. The second-level Poisson equation models heterogeneity in the total number of latent events across observational units.

A key property of the pogit model is that the latent count admits a simple predictive distribution, as stated in the following property:

**Property 1.** 
*For each observational unit i=1,…,N in a pogit model, $\left(n_{i}-y_{i}|y_{i},\theta_{i},\lambda_{i}\right)∼\mathrm{Poisson}\left(E_{i}\lambda_{i}\left(1-\theta_{i}\right)\right)$.*

This result follows from the Poisson thinning property (see, e.g., [14]) and plays an important role in the EM algorithm developed below.

### 2.2. Regression Structure

Both the reporting probability and the latent intensity are linked to covariates through separate regression specifications:

$$\begin{array}{cc} \theta_{i}=\mathrm{sigmoid}(\eta_{i1}), & \;i=1,\ldots ,N,\\ \lambda_{i}=\exp(\eta_{i2}),\; & \;j=1,2, \end{array}$$

where $\eta_{ij}=x_{ij}^{\prime }\beta_{j}$ denotes the linear predictor associated with component *j* for observational unit *i*, $x_{ij}\in R^{K_{j}}$ is a column vector of observed covariates (with $x_{ij}^{\prime }$ denoting its transpose), $\beta_{j}\in R^{K_{j}}$ is the corresponding vector of regression coefficients, $K_{j}$ denotes the number of covariates entering component *j*, and $\mathrm{sigmoid}\left(x\right)\equiv 1/\left(1+e^{-x}\right)$ is the sigmoid (or expit) function. This specification allows the reporting mechanism and the latent intensity to depend on distinct, possibly overlapping, covariate sets. Such flexibility is essential in applications such as under-reporting correction and share-of-wallet estimation. Moreover, the chosen link functions yield interpretable parameters and facilitate likelihood augmentation.

Throughout the paper, the model is defined in terms of the natural parameters $\left(\theta_{i},\lambda_{i}\right)$, while many derivations and algorithmic steps are expressed in terms of the associated linear predictors $\left(\eta_{i1},\eta_{i2}\right)$, where $\theta_{i}=\mathrm{sigmoid}\left(\eta_{i1}\right)$ and $\lambda_{i}=\exp\left(\eta_{i2}\right)$. This reparameterization is purely deterministic and one-to-one. For notational simplicity and computational convenience, we work with whichever representation is more appropriate in a given context, and the mapping between the two is made explicit whenever it is used.

**Figure 1:**
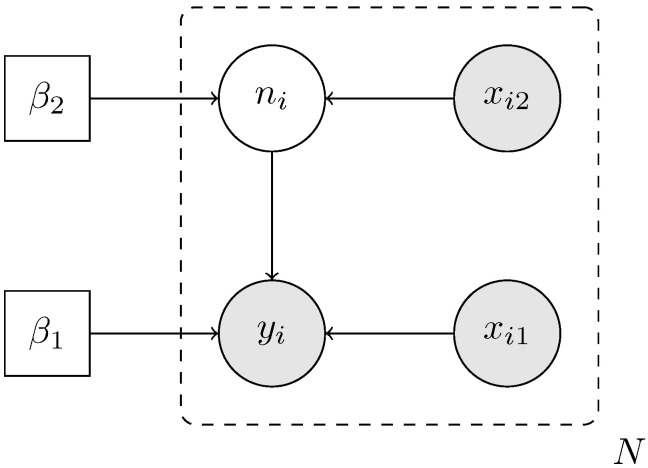
Graphical representation of the pogit model. Circles denote random variables and arrows indicate conditional dependence. Shaded nodes represent observed quantities.

Figure 1 provides a graphical representation of the model using plate notation [15]. Regression coefficients $\beta ={\left(\beta_{1}^{\prime },\beta_{2}^{\prime }\right)}^{\prime }$ are shared across observational units, while $n_{i}$ and $y_{i}$ are unit-specific latent and observed variables.

### 2.3. Observed Likelihood and Identification

Marginalizing over the latent count $n_{i}$ yields

$$\begin{array}{c} \left(y_{i}|\theta_{i},\lambda_{i}\right)\overset{ind}{∼}\mathrm{Poisson}\left(E_{i}\theta_{i}\lambda_{i}\right),\;i=1,\ldots ,N, \end{array}$$

see, e.g., Kingman [14]. The observed-data likelihood, therefore, depends on the parameters only through the product $\theta_{i}\lambda_{i}$. As a consequence, without additional structure, the reporting probability and the latent intensity are not separately identified. In particular, for any c>0, the transformation $\theta_{i}\mapsto c\theta_{i}$ and $\lambda_{i}\mapsto \lambda_{i}/c$ leaves the likelihood unchanged.

Identification is restored by the regression structure, which links $\theta_{i}$ and $\lambda_{i}$ to covariates through distinct linear predictors. Under mild regularity conditions, this structure ensures local identification of the parameter vector β, that is, that there is a neighborhood of the true β without observationally equivalent parameter values (in the sense of producing the exact same likelihood). The identification result is not merely technical. Local identification is a necessary condition for the observed-data likelihood to admit a locally unique maximizer in a neighborhood of the true parameter value, thereby ruling out flat ridges generated by observationally equivalent parameterizations. This property underpins meaningful likelihood-based inference by ensuring that distinct parameter values correspond to distinct data-generating processes in a local neighborhood. While local identification does not preclude the presence of nearly flat regions of the likelihood, it guarantees local uniqueness of the true parameter, which is a necessary condition for well-defined EM-based estimation. A formal definition of local identification and the associated regularity concepts are provided in Appendix A.

**Theorem 1.** 
*Let i=1,…,N, and let $x_{i}^{\prime }=\left(x_{i1}^{\prime },x_{i2}^{\prime }\right)$ and $z_{i}^{\prime }=\left(\left(1-\theta_{i}\right)x_{i1}^{\prime },x_{i2}^{\prime }\right)$. Suppose ${\left\{\left(y_{i},x_{i}\right)\right\}}_{i=1}^{N}$ is an i.i.d. sequence such that $E[z_{i}z_{i}^{\prime }]$ is positive definite. Then the pogit parameters are locally identified.*

**Proof.**  See Appendix A.    □

**Remark 1**  (Overlap and weak identification)**.** *Local identification may hold even when $x_{i1}$ and $x_{i2}$ overlap. However, strong overlap or collinearity renders the Fisher information matrix nearly singular, leading to weak identification in finite samples. Applied work, therefore, often relies on distinct covariates across hierarchical levels (see, e.g., [2]), or incorporates auxiliary data in which the counts $n_{i}$ are observed (see, e.g., [8]).*

**Remark 2** 
(Local versus global identification)**.** *Theorem 1 establishes local, but not global, identification. As shown by Brennan et al. [16], when covariates entering the intensity equation are a subset of those entering the reporting equation, the model may be locally identified while remaining globally unidentified. Common remedies include reducing covariate overlap, incorporating auxiliary observations on latent counts, or imposing sign restrictions informed by expert judgment.*

From a computational perspective, the observed-data likelihood is highly nonlinear in β. Direct maximization requires numerical optimization and becomes increasingly costly as the sample size and covariate dimension grow. Moreover, the multiplicative interaction between $\theta_{i}$ and $\lambda_{i}$ often leads to instability in large samples. These features motivate the search for an augmented representation with simpler structure.

### 2.4. A Naive EM Algorithm

The hierarchical formulation naturally suggests an EM algorithm based on the augmented likelihood $p\left(y,n|\beta \right)=\prod_{i}p\left(y_{i}|n_{i},\theta_{i}\right)p\left(n_{i}|\lambda_{i}\right)$, that is

$$\begin{array}{c} p\left(y,n|\beta \right)=\prod_{i}\mathrm{Bin}\left(y_{i}|n_{i},\theta_{i}\right)\mathrm{Poisson}\left(n_{i}|E_{i}\lambda_{i}\right). \end{array}$$

Given a current iterate $\beta^{\left(t\right)}$, such an algorithm replaces $n_{i}$ by $E[n_{i}|y_{i},\beta^{\left(t\right)}]$ in the E-step and updates $\beta ={\left(\beta_{1}^{\prime },\beta_{2}^{\prime }\right)}^{\prime }$ via binomial and Poisson regressions in the M-step. While formally valid, this approach is computationally unattractive: both regressions require iterative solvers, so each EM iteration contains nested optimization loops. As a result, the procedure scales poorly and offers little computational advantage over direct likelihood maximization.

The key insight of this article is that augmenting only with the latent counts $n_{i}$ is *insufficient* to obtain a scalable EM algorithm, because the resulting complete likelihood remains non-quadratic in the regression parameters.

To overcome this limitation, we exploit the fact that both components of the pogit model admit conditionally Gaussian representations under suitable augmentations. The binomial component admits an exact Polya-Gamma augmentation, while the Poisson component can be accurately approximated by a negative-binomial pmf that also yields a Polya-Gamma augmentation.

Introducing Polya-Gamma variables in addition to the latent counts renders the complete log-likelihood quadratic in $\left(\beta_{1},\beta_{2}\right)$. The resulting EM algorithm features closed-form E-step expectations and M-steps that reduce to weighted least-squares problems with explicit solutions. This structure yields low per-iteration cost and naturally accommodates large-scale extensions. The construction of this augmented likelihood and the corresponding EM updates are developed in the next section.

## 3. An Improved EM Algorithm

In this section, we present a scalable expectation–maximization (EM) algorithm for the pogit model that exploits Polya-Gamma augmentation to obtain a quadratic complete–data log-likelihood and closed-form updates in both the E- and M-steps. As mentioned in Section 2, the algorithm is based on the Polya-Gamma distribution, so we start by explaining this distribution and its key properties.

### 3.1. The Polya-Gamma Distribution

The Polya-Gamma distribution was introduced by Polson et al. [11] as part of a new data augmentation for logistic regression models. A random variable w≥0 is said to follow a Polya-Gamma distribution with parameters (b,c), denoted w∼PG(b,c), where b>0 and c∈R, if it admits the representation

$$\begin{array}{c} w\;\overset{d}{=}\;\frac{1}{2\pi^{2}}\sum_{k=1}^{\infty }\frac{g_{k}}{{\left(k-1/2\right)}^{2}+c^{2}/\left(4\pi^{2}\right)}, \end{array}$$

where ${\left\{g_{k}\right\}}_{k\geq 1}$ are independent Gamma(b,1) random variables.

The Polya-Gamma distribution has two key properties.

**Property 2.** 
*For any a,ψ∈R and b>0,*

$$\begin{array}{cc} \frac{{\left(e^{\psi }\right)}^{a}}{{\left(1+e^{\psi }\right)}^{b}} & =2^{-b}\int_{0}^{\infty }\exp\left(\kappa \psi -w\psi^{2}/2\right)p\left(w|b,0\right)\;dw, \end{array}$$

*where κ=a−b/2 and w∼PG(b,0).*

This property will be useful for our EM algorithm, as it transforms binomial and negative-binomial likelihoods into Gaussian kernels conditional on the latent variable *w*.

**Property 3.** 
*Let w∼PG(b,c). Then, E[w]=(b/4)tanhc(c/2) for any c>0 and E[w]=b/4 otherwise, where tanhc(x):=tanh(x)/x.*

This property will be particularly convenient for our EM algorithm, as it will allow the E-step to be computed analytically without numerical integration.

### 3.2. The Augmented Likelihood

We now derive our likelihood augmentation. The central idea is to augment the pogit model so that the complete-data log-likelihood becomes quadratic in the regression parameters $\left(\beta_{1},\beta_{2}\right)$, yielding closed-form updates in the M-step and eliminating inner optimization loops. For ease of exposition, we present the main steps of the likelihood augmentation here; the full derivation is deferred to Appendix B.

Consider the *i*th contribution to the naive complete likelihood,

$$\begin{array}{c} p(n_{i},y_{i}|\beta )=\mathrm{Bin}\left(y_{i}|n_{i},\theta_{i}\right)\mathrm{Poisson}\left(n_{i}|E_{i}\lambda_{i}\right). \end{array}$$

We treat the two components separately.

Before deriving the augmented likelihood contributions, recall that $\eta_{i1}=x_{i1}^{\prime }\beta_{1}$ and $\eta_{i2}=x_{i2}^{\prime }\beta_{2}$ denote the linear predictors associated with the binomial (reporting) and latent intensity components, respectively.

#### 3.2.1. Binomial Component

Using the Polya-Gamma identity in Property 2 and rearranging terms, the binomial likelihood can be written as

$$\begin{array}{c} \mathrm{Bin}(y_{i}|n_{i},\theta_{i})\propto E_{w_{i1}}\exp\left(\kappa_{i1}\eta_{i1}-w_{i1}\eta_{i1}^{2}/2\right), \end{array}$$

where $E_{w_{i1}}$ denotes expectation with respect to $w_{i1}∼\mathrm{PG}\left(n_{i},0\right)$, $\kappa_{i1}=y_{i}-n_{i}/2$, and the symbol ∝ denotes equality up to a multiplicative constant independent of the parameters of interest. Therefore, conditional on $w_{i1}$, the binomial likelihood contribution can be expressed as an exponential quadratic form in the linear predictor $\eta_{i1}$, that is, as a Gaussian kernel (up to a normalizing constant).

#### 3.2.2. Poisson Component

Unlike the binomial likelihood, the Poisson likelihood does not admit an exact Polya-Gamma representation. However, it is well-known that the $\mathrm{NegBin}(r_{i},E_{i}\lambda_{i}/\left(r_{i}+E_{i}\lambda_{i}\right))$ distribution converges in distribution to the $\mathrm{Poisson}(E_{i}\lambda_{i})$ distribution as $r_{i}\to \infty$ (see, e.g., [14]). Unlike the binomial likelihood, the Poisson likelihood does not admit an exact Polya-Gamma representation. We, therefore, approximate it using a negative-binomial distribution with parameter $r_{i}\gg 0$, where $r_{i}$ controls the accuracy of the approximation.

Hence, we can approximate

$$\begin{array}{c} \mathrm{Poisson}(n_{i}|E_{i}\lambda_{i})\approx \mathrm{NegBin}(n_{i}|r_{i},E_{i}\lambda_{i}/\left(r_{i}+E_{i}\lambda_{i}\right)), \end{array}$$

for some $r_{i}\gg 0$. Using Property 2 and simplifying, we obtain

$$\begin{array}{c} \mathrm{Poisson}(n_{i}|E_{i}\lambda_{i})\propto E_{w_{i2}}\exp(\kappa_{i2}\eta_{i2}-w_{i2}\eta_{i2}^{2}/2-\frac{w_{i2}}{2}\;{\left(\log(r_{i}/E_{i})\right)}^{2}, \end{array}$$

where $E_{w_{i2}}$ denotes expectation with respect to $w_{i2}∼\mathrm{PG}\left(n_{i}+r_{i},0\right)$, $\kappa_{i2}=\left(n_{i}-r_{i}\right)/2+w_{i2}\log\left(r_{i}/E_{i}\right)$. Therefore, conditional on $w_{i2}$, the approximated Poisson likelihood can be expressed as an exponential quadratic form in the linear predictor $\eta_{i2}$, that is, as a Gaussian kernel (up to a normalizing constant). This representation is key to obtaining closed-form updates in the M-step.

**Remark 3.** 
*The use of a negative-binomial approximation to enable Polya-Gamma augmentation for Poisson models was first proposed by D’Angelo et al. [13] in the context of Bayesian Poisson regression, and shown to improve computational efficiency relative to earlier approaches.*

**Remark 4.** 
*Although the proposed augmentation is based on an approximation rather than an exact identity, its quality is fully controllable: accuracy can be made arbitrarily high by increasing $r_{i}$, with the only practical limitation being numerical stability. In practice, even moderate values of $r_{i}$ already yield excellent approximations; in our experiments, $r_{i}=100$ produced indistinguishable parameter estimates. For fixed $r_{i}$, the proposed procedure is an exact EM algorithm for a well-defined approximating likelihood based on a negative-binomial representation of the Poisson component, and this likelihood converges pointwise to the pogit likelihood as $r_{i}\to \infty$.*

#### 3.2.3. Augmented Log-Likelihood

Introducing the aforementioned $w_{ij}$’s to the model, we obtain the following augmented log-likelihood:

$$\begin{array}{c} \log p(y,n,w|\beta )≐\sum_{ij}\left(\kappa_{ij}\eta_{ij}-w_{ij}\eta_{ij}^{2}/2\right)-\sum_{i}w_{i2}{\left(\log(r_{i}/E_{i})\right)}^{2}/2, \end{array}$$

where ≐ denotes equality up to additive constants that do not depend on the parameters of interest (in this case, β).

### 3.3. EM Steps

Let $\beta^{\left(t\right)}$ denote the current iterate of β. As with any EM algorithm, our procedure updates this value following two general steps:

- E-step: compute the *Q*-function, $Q\left(\beta \right):=E[\log p\left(y,n,w|\beta \right)|y,\beta^{\left(t\right)}]$.
- M-step: update β by maximizing the *Q*-function.

As we shall see, both steps admit closed-form expressions, leading to a simple and efficient EM algorithm.

#### 3.3.1. E-Step

First, note that Q(β) depends on β only through η. In particular,

$$\begin{array}{c} Q(\beta )≐\sum_{ij}\left({\hat{\kappa }}_{ij}\eta_{ij}-{\hat{w}}_{ij}\eta_{ij}^{2}/2\right)-\sum_{i}{\hat{w}}_{i2}{\left(\log(r_{i}/E_{i})\right)}^{2}/2, \end{array}$$

where $\hat{a}=E\left[a|y,\beta^{\left(t\right)}\right]$ denotes the conditional expectation of any random variable *a* (for instance, $\kappa_{ij}$). We now explain how to compute ${\hat{\kappa }}_{ij}$ and ${\hat{w}}_{ij}$.

*Computing ${\hat{\kappa }}_{ij}$.* This is straightforward as it is linear in (n,w):

$$\begin{array}{cc} {\hat{\kappa }}_{i1} & =y_{i}-{\hat{n}}_{i}/2,\\ {\hat{\kappa }}_{i2} & =\left({\hat{n}}_{i}-r_{i}\right)/2+{\hat{w}}_{i2}\log\left(r_{i}/E_{i}\right). \end{array}$$

*Computing ${\hat{n}}_{i}$.* This is also straightforward. Property 1 implies that $E[n_{i}|y,\beta ]$ is equal to $y_{i}+E_{i}\left(1-\theta_{i}\right)\lambda_{i}=y_{i}+E_{i}\exp\left(\eta_{i2}\right)/\left(1+\exp\left(\eta_{i1}\right)\right)$, so evaluating at $\beta =\beta^{\left(t\right)}$ gives

$$\begin{array}{c} {\hat{n}}_{i}=y_{i}+E_{i}\exp\left({\hat{\eta }}_{i2}\right)/\left(1+\exp\left({\hat{\eta }}_{i1}\right)\right). \end{array}$$

*Computing ${\hat{w}}_{ij}$.* This is more challenging, but it is well-known that

- $\left(w_{i1}|y,n,\beta \right)∼\mathrm{PG}\left(n_{i},\eta_{i1}\right)$ [11].
- $\left(w_{i2}|y,n,\beta \right)∼\mathrm{PG}\left(n_{i}+r_{i},\eta_{i2}-\log\left(r_{i}/E_{i}\right)\right)$ [13].

Hence, using Property 3 and the law of iterated expectations, we obtain

$$\begin{array}{cc} {\hat{w}}_{i1} & =0.25{\hat{n}}_{i}\mathrm{tanhc}\left(0.5{\hat{\eta }}_{i1}\right),\\ {\hat{w}}_{i2} & =0.25\left({\hat{n}}_{i}+r_{i}\right)\mathrm{tanhc}\left(0.5\left({\hat{\eta }}_{i2}-\log\left(r_{i}/E_{i}\right)\right)\right). \end{array}$$

In summary, Q(β) has a closed-form expression.

**Remark 5.** 
*In order to apply the E-step successfully, the functions sigmoid(·) and tanhc(·) must be evaluated in a numerically stable way. In particular, we use*

$$\begin{array}{cc} \mathrm{sigmoid}(x) & =\left\{\begin{array}{cc} \exp(x)/(1+\exp(x)), & \mathrm{if}\;x<0\\ 1/(1+\exp(-x)), & \mathrm{otherwise} \end{array}\right. \end{array}$$

*and*

$$\begin{array}{cc} \mathrm{tanhc}(x) & =\left\{\begin{array}{cc} 1-x^{2}/3+2x^{4}/15-17x^{6}/315, & \mathrm{if}\;|x|<\epsilon \\ \tanh(x)/x, & \mathrm{otherwise} \end{array}\right. \end{array}$$

*In our experiments, $\epsilon =10^{-4}$ worked fine.*

**Remark 6.** 
*Exploiting the closed-form mean of the Polya-Gamma distribution within an expectation-maximization framework was first proposed by Scott et al. [12] in the context of logistic regression, where it was shown to yield substantial computational gains relative to naive optimization strategies.*

#### 3.3.2. M-Step

As Q(β) is quadratic in β, maximization yields closed-form solutions. For j=1,2, let $X_{j}$ denote the design matrix collecting the covariates $x_{ij}^{\prime }$ associated with component *j* across all observational units. By standard least-squares theory, the solution is given by

$$\begin{array}{c} \beta_{j}^{\left(t+1\right)}={\left(X_{j}^{\prime }{\hat{W}}_{j}X_{j}\right)}^{-1}X_{j}^{\prime }{\hat{\kappa }}_{j},\;j=1,2 \end{array}$$

where ${\hat{W}}_{j}$ is a diagonal matrix with entries ${\hat{w}}_{ij}$.

In this way, each EM iteration reduces to two independent weighted least-squares problems that require no any inner iterative procedure. In addition, each update has a structure particularly well suited for parallel and mini-batch extensions.

**Remark 7.** 
*The Polya-Gamma augmentation used here is closely related to constructions commonly exploited in variational Bayes (VB) methods (see, e.g., [17]) for logistic models [18]. The present approach, however, differs in both objective and implementation. Our algorithm is an expectation-maximization procedure that directly targets the observed-data likelihood of the pogit model, rather than a variational lower bound, and imposes no factorization assumptions on latent variables. All conditional expectations in the E-step are computed exactly under the augmented model, and stochastic approximation is used solely to scale the computation of sufficient statistics in large samples. As a result, the method retains the likelihood-based interpretation and asymptotic properties of maximum-likelihood estimation, while achieving computational efficiency comparable to VB approaches.*

## 4. Computational Enhancements

The EM algorithm introduced in Section 3 has two key computational advantages: each iteration has low cost due to closed-form updates, and the algorithm admits a simple representation in terms of sufficient statistics. Nevertheless, two practical challenges remain in large-scale applications. First, when the sample size *N* is very large, even inexpensive full-batch iterations can become costly. Second, like most fixed-point algorithms, EM may converge slowly when the likelihood surface is flat or parameters are weakly identified.

In this section, we address these challenges using two complementary strategies. To scale the algorithm to massive datasets, we develop a mini-batch EM scheme based on Robbins–Monro stochastic approximation. To accelerate convergence (even in samples of moderate size), we combine the EM updates with a quasi-Newton extrapolation technique known as SQUAREM. These enhancements target distinct computational bottlenecks and can be used independently or in combination.

### 4.1. Mini-Batch EM via Robbins–Monro

To reduce computational cost when *N* is large, we adopt an online EM approach based on stochastic approximation [19], following the general framework of Cappé and Moulines [20]. The key observation is that the EM algorithm derived in Section 3 depends on the data only through additive sufficient statistics, making it particularly well suited for mini-batching.

Recall that the M-step for component j∈{1,2} depends on the statistics

$$\begin{array}{cc} S_{j}^{\left(t\right)}=X_{j}^{\prime }{\hat{W}}_{j}X_{j},\; & s_{j}^{\left(t\right)}=X_{j}^{\prime }{\hat{\kappa }}_{j}. \end{array}$$

Given a random mini-batch $B_{t}\subseteq \left\{1,\ldots ,N\right\}$, unbiased estimators are

$$\begin{array}{cc} {\hat{S}}_{j}^{\left(t\right)}=\frac{N}{|B_{t}|}\sum_{i\in B_{t}}{\hat{w}}_{ij}x_{ij}x_{ij}^{\prime },\; & {\hat{s}}_{j}^{\left(t\right)}=\frac{N}{|B_{t}|}\sum_{i\in B_{t}}{\hat{\kappa }}_{ij}x_{ij}, \end{array}$$

where randomness arises solely from subsampling.

A naive approach would replace $\left(S_{j}^{\left(t\right)},s_{j}^{\left(t\right)}\right)$ directly with their mini-batch counterparts. However, although unbiased, these estimators exhibit persistent sampling noise that prevents convergence of the resulting EM iterations. To stabilize the procedure, Cappé and Moulines [20] propose updating the sufficient statistics using diminishing step sizes, that is

$$\begin{array}{c} S_{j}^{\left(t+1\right)}=\left(1-\gamma_{t}\right)S_{j}^{\left(t\right)}+\gamma_{t}{\hat{S}}_{j}^{\left(t\right)},\\ s_{j}^{\left(t+1\right)}=\left(1-\gamma_{t}\right)s_{j}^{\left(t\right)}+\gamma_{t}{\hat{s}}_{j}^{\left(t\right)}, \end{array}$$

where $S_{j}^{\left(0\right)}=0$, $s_{j}^{\left(0\right)}=0$, and the step size sequence ${\left\{\gamma_{t}\right\}}_{t\geq 1}$ shrinks in such a way that $\sum_{t}\gamma_{t}=\infty$ and $\sum_{t}\gamma_{t}^{2}<\infty$.

In practice, we use

$$\begin{array}{c} \gamma_{t}=\left\{\begin{array}{cc} c, & t\leq t_{\mathrm{burn}},\\ c/{\left(t-t_{\mathrm{burn}}+t_{0}\right)}^{a}, & t>t_{\mathrm{burn}}, \end{array}\right. \end{array}$$

with c>0, a∈(1/2,1), and $t_{\mathrm{burn}},t_{0}>0$. The initial constant phase stabilizes early iterations, while the polynomial decay ensures asymptotic convergence.

To further reduce variance and improve finite-sample performance, we apply *Polyak averaging* [21,22] to the parameter iterates after burn-in. Specifically, the reported estimator is

$$\begin{array}{c} \bar{\beta }={\left(T-t_{\mathrm{burn}}\right)}^{-1}\sum_{t>t_{\mathrm{burn}}}\beta^{\left(t\right)}, \end{array}$$

which achieves optimal asymptotic variance for stochastic approximation schemes [20].

This mini-batch EM procedure preserves the structure of the exact Polya-Gamma EM updates while reducing per-iteration complexity to $O(|B_{t}|K_{j}^{2})$, independent of the total sample size. As a result, the method scales naturally to datasets with millions of observations.

**Remark 8.** 
*Mini–batch EM algorithms based on stochastic approximation may require a large number of iterations and can exhibit substantial variability in the raw parameter iterates. For this reason, we recommend using a sufficiently large iteration budget (at least 10,000 iterations in our experiments) and assessing convergence in terms of the Polyak–averaged estimator $\bar{\beta }$ rather than the instantaneous iterate $\beta^{\left(t\right)}$. This practice stabilizes inference and aligns with standard recommendations in stochastic approximation.*

**Remark 9.** 
*Unlike full-batch EM, mini-batch EM does not guarantee a monotone increase in the observed-data likelihood. While monotonicity can in principle be enforced via safeguarding steps, our simulation experiments indicate that the proposed algorithm exhibits stable behavior without such modifications. For this reason, and to preserve computational simplicity, we adopt the unsafeguarded version in our implementation.*

### 4.2. Quasi-Newton Acceleration via SQUAREM

Even in full-batch settings, EM algorithms may converge slowly when the likelihood surface is flat or parameters are weakly identified. To accelerate convergence, we complement our method with a quasi-Newton extrapolation technique known as SQUAREM [23].

Let M(β) denote the EM update mapping induced by one full E- and M-step, so that the standard EM iteration is $\beta^{\left(t+1\right)}=M\left(\beta^{\left(t\right)}\right)$. SQUAREM treats EM as a fixed-point iteration and constructs an accelerated update by extrapolating along the EM trajectory. Starting from $\beta^{\left(t,0\right)}:=\beta^{\left(t\right)}$, define

$$\begin{array}{cc} \beta^{\left(t,h\right)} & =M(\beta^{\left(t,h-1\right)}),\;h=1,2,\\ r & =\beta^{\left(t,1\right)}-\beta^{\left(t,0\right)},\\ v & =\beta^{\left(t,2\right)}-\beta^{\left(t,1\right)}-r. \end{array}$$

Here, *r* represents the first-order displacement induced by EM, while *v* captures curvature by measuring deviations from linearity along the EM path.

The accelerated update is then given by

$$\begin{array}{c} \beta_{\mathrm{SQ}}^{\left(t+1\right)}=\beta^{\left(t\right)}-2\alpha r+\alpha^{2}v, \end{array}$$

where the step size α is chosen to approximately minimize the norm of the residual. Following Varadhan and Roland [23], we set

$$\begin{array}{c} \alpha =-{∥r∥}_{2}/{∥v∥}_{2}. \end{array}$$

A safeguarding step ensures monotonicity of the observed log-likelihood, reverting to the standard EM update whenever the extrapolated iterate decreases the likelihood.

SQUAREM is particularly well-suited to our Polya-Gamma-based EM algorithm. First, each EM iteration is deterministic and inexpensive, making the cost of additional EM evaluations negligible relative to the gains in convergence speed. Second, the M-step consists of weighted least-squares updates, which vary smoothly with the parameters and favor quasi-Newton extrapolation. Third, the method is entirely generic and requires no modification of the underlying EM structure.

In our empirical experiments, SQUAREM substantially reduces the number of EM iterations required to reach convergence, often by an order of magnitude, while preserving numerical stability. For this reason, we recommend SQUAREM as the default acceleration strategy when fitting the pogit model in moderate to large samples.

## 5. Simulation Study

This section evaluates the proposed EM algorithm along three complementary dimensions. First, we study its finite-sample estimation behavior under moderate sample sizes, focusing on signal recovery and dispersion across Monte Carlo replications. Second, we assess numerical robustness with respect to the negative-binomial approximation parameter underlying the Polya-Gamma augmentation. Third, we examine computational scalability in large samples and quantify the gains achieved by standard acceleration techniques. Together, these experiments are designed to validate the statistical accuracy, numerical stability, and practical scalability of the proposed estimation framework.

**Remark 10.** 
*All simulations were conducted on a desktop computer equipped with an Intel® Core™ i7-9750H CPU (6 cores, 2.60GHz) and 16 GB of RAM, running Windows 11 (64-bit).*

### 5.1. Finite-Sample Estimation Behavior

We begin by examining the finite-sample behavior of the EM estimator in moderate sample sizes. In all scenarios, each model component includes nine covariates (i.e., $K_{1}=K_{2}=9$). For each observational unit *i*, the covariate vectors are generated independently according to $x_{ij}∼N\left(0_{9},I_{9}/3\right)$. The scaling factor 1/3 is chosen so that, if all regression coefficients are set equal to one, the resulting linear predictors $\eta_{ij}=x_{ij}^{\prime }\beta_{j}$ have variance of moderate magnitude. This normalization keeps the linear predictors within a numerically stable range for the logistic and exponential link functions throughout the simulations.

The true parameter vectors are fixed at

$$\begin{array}{cccc} \beta_{1} & =(1,2,0,\ldots ,0),\; & \beta_{2} & =(2,1,0,\ldots ,0), \end{array}$$

so that only the first two coefficients in each vector are nonzero. This design induces a sparse signal structure and allows us to assess signal recovery.

Throughout the simulation study, the exposure term is fixed at $E_{i}\equiv 1$ for all *i*. We consider two sample sizes: N=500 (Scenario 1) and N=1000 (Scenario 2). For each scenario, R=100 independent datasets are generated from the data-generating process described above. In each replication, the EM algorithm is initialized randomly and iterated until convergence, with a maximum of 1000 iterations allowed. Convergence is declared when the maximum relative change across all parameter components between successive iterations falls below $10^{-4}$.

**Figure 2:**
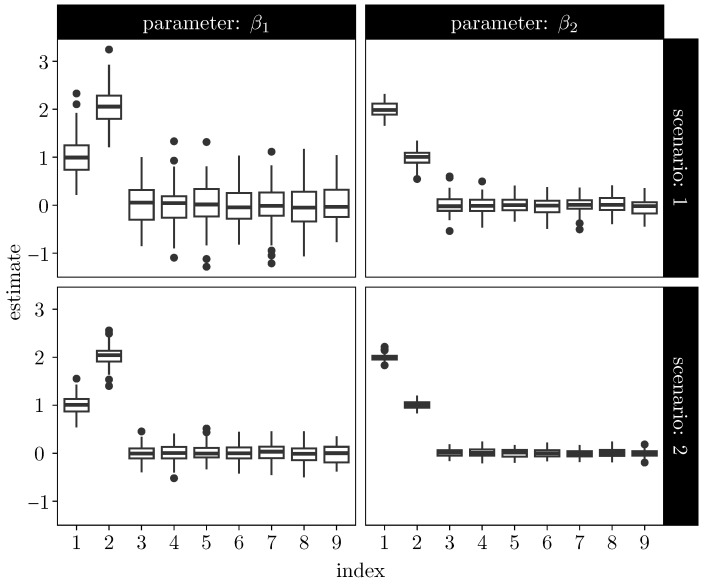
Boxplots of EM estimates across R=100 Monte Carlo replications for the coefficients $\beta_{1}=\left(1,2,0,\ldots ,0\right)$ (**left**) and $\beta_{2}=\left(2,1,0,\ldots ,0\right)$ (**right**) for two sample sizes: N=500 (**top**) and N=1000 (**bottom**). The horizontal axis corresponds to the index of the regression coefficient within each parameter vector (i.e., the *k*th component of $\beta_{j}$). Coefficients that are truly zero concentrate near zero, while nonzero coefficients are recovered with reduced dispersion as sample size increases.

Figure 2 summarizes the resulting Monte Carlo distributions of the EM estimates. The nonzero coefficients in $\beta_{1}$ and $\beta_{2}$ are accurately recovered in both scenarios, while coefficients that are truly zero remain tightly concentrated around zero. Increasing the sample size from 500 to 1000 leads to a visible reduction in dispersion across replications, indicating improved estimator concentration in larger samples.

### 5.2. Robustness to the Approximation Parameter

Having established stable finite-sample behavior, we next examine robustness with respect to the tuning parameter *r* controlling the accuracy of the negative-binomial approximation used to obtain a Polya-Gamma augmentation of the Poisson component. While larger values of *r* yield a closer approximation to the Poisson likelihood, excessively large choices may introduce unnecessary numerical overhead and potential numerical issues. Evaluating sensitivity to this parameter is, therefore, important for practical implementation.

**Figure 3:**
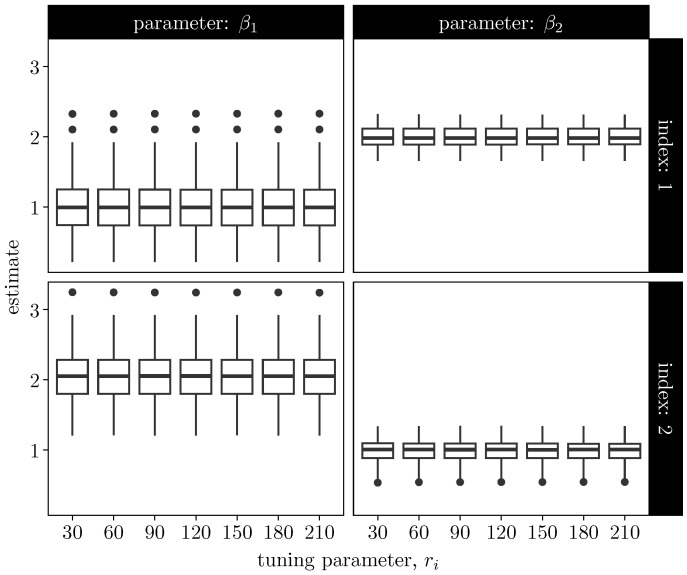
Sensitivity of EM estimates to the negative-binomial tuning parameter $r_{i}$. Boxplots are shown for selected coefficients (indices 1 and 2, corresponding to the first two components of each coefficient vector) of $\beta_{1}$ and $\beta_{2}$ across values $r_{i}\in \left\{30,60,\ldots ,210\right\}$. No systematic changes in the estimates are observed as $r_{i}$ varies.

Figure 3 reports Monte Carlo boxplots for selected coefficients (indices 1 and 2) of $\beta_{1}$ and $\beta_{2}$ across values r∈{30,60,…,210}, with $r_{i}\equiv r$ for all *i*. Across all panels, the empirical distributions remain stable as *r* varies. While Monte Carlo variability is present, no systematic shifts or trends are observed as *r* increases from relatively small to moderately large values.

These results indicate that the proposed EM algorithm is not unduly sensitive to the precise choice of the tuning parameter, provided that *r* is chosen sufficiently large to yield an accurate quadratic approximation of the Poisson component. In particular, moderate values such as r=100, which we adopt as a default in subsequent experiments, already yield estimation behavior comparable to that observed for larger values.

**Remark 11.** 
*To complement the results of this particular experiment, we included three additional figures in Appendix C, showing the sensitivities of the estimates’ standard errors, the observed likelihood and the effective number of iterations to r. Together, these figures show that the effective number of iterations grows with r, but standard errors and the log-likelihood tend to stabilize at r∼100. These supplementary results strengthen our practical recommendation of r=100.*

### 5.3. Computational Scalability and Acceleration

Having established finite-sample accuracy and numerical robustness, we now turn to computational performance. These experiments focus on scalability in large samples and are designed to assess whether the proposed EM algorithm and its accelerated variants remain practical in regimes where existing pogit implementations become computationally prohibitive.

We consider three complementary comparisons. First, we benchmark the proposed deterministic EM algorithm against direct maximum-likelihood (ML) optimization of the observed-data likelihood. Second, we evaluate the impact of mini-batch estimation via Robbins–Monro updates on runtime scalability. Third, we assess the gains from quasi-Newton acceleration using SQUAREM. In all cases, methods target the same likelihood and are run under identical initialization/stopping rules. For the Robbins–Monro mini-batch EM variant, stepsize sequences were chosen according to standard diminishing-stepsize conditions; all tuning parameters are reported in Supplementary File S1.

**Figure 4:**
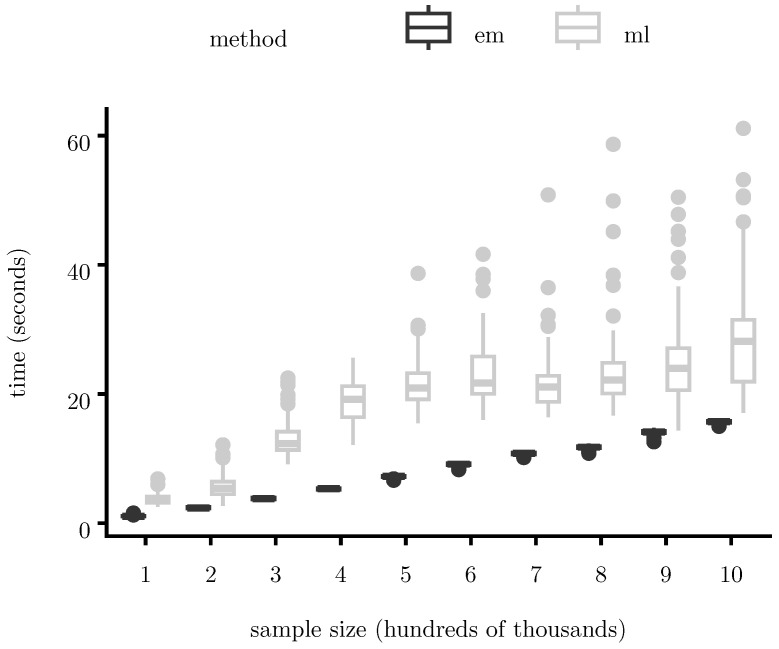
Runtime scaling of the proposed EM algorithm as a function of sample size. Across all sample sizes, EM is consistently faster than direct ML optimization, with the difference becoming more pronounced as *N* increases. For both algorithms, we allowed a maximum of 1000 iterations, and convergence was declared if the maximum relative change across all parameter components between successive iterations falls below $10^{-4}$.

**Figure 5:**
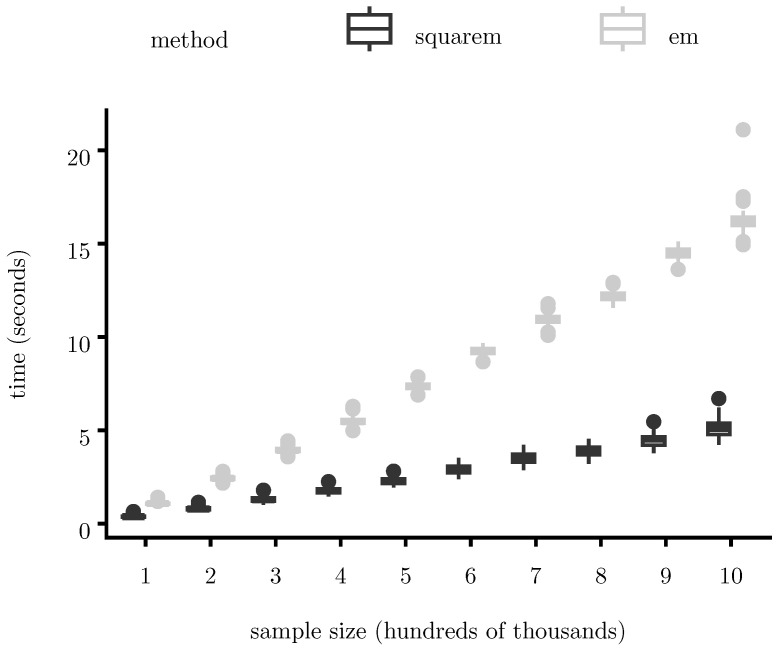
Runtime comparison between standard EM and SQUAREM-accelerated EM. SQUAREM applies safeguarded squared extrapolation and yields substantial runtime reductions for large samples. For both algorithms, we allowed a maximum of 1000 iterations, and convergence was declared if the maximum relative change across all parameter components between successive iterations falls below $10^{-4}$.

**Figure 6:**
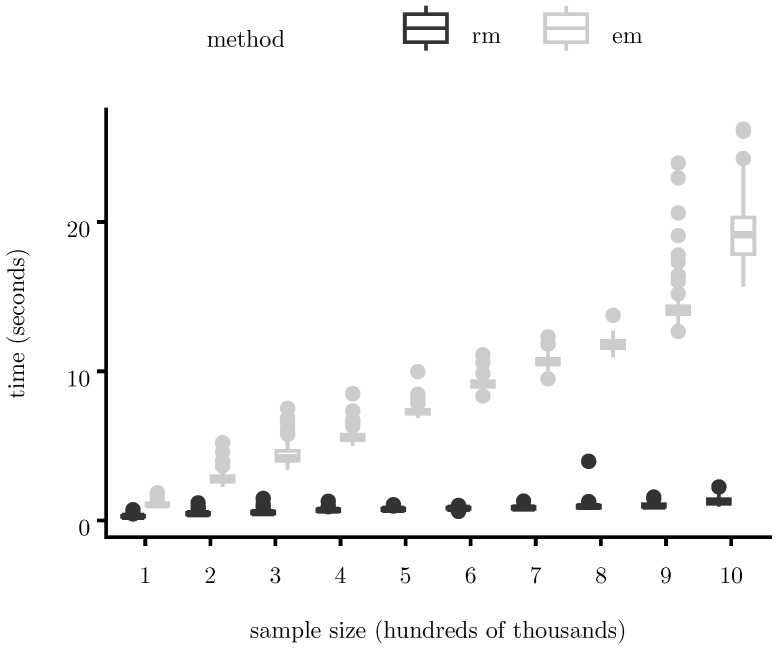
Runtime comparison between full-batch EM and Robbins–Monro mini-batch EM as a function of sample size. All methods use identical initialization and stopping rules. For the first algorithm, we allowed a maximum of 1000 iterations, and convergence was declared if the maximum relative change across all parameter components between successive iterations falls below $10^{-4}$. For the second estimator, we allowed a maximum of 10,000 iterations, and convergence was declared if the maximum relative change across all parameter components between successive Polyak-averages falls below $10^{-4}$.

Figure 4 and Figure 5 report total runtime (measured in seconds) as a function of sample size (measured in hundreds of thousands of observations). Figure 4 compares the proposed EM algorithm with direct ML optimization (implemented in Julia using BFGS via the R function stats::optim(); Figure 5 compares standard EM with its SQUAREM-accelerated version and Figure 6 compares full-batch EM with a Robbins–Monro mini-batch variant.

Across all methods, runtime grows approximately linearly with sample size over the ranges considered. The proposed EM algorithm consistently outperforms direct ML optimization, with a consistent runtime advantage across all sample sizes considered. The Robbins–Monro variant further reduces runtime in very large samples by lowering per-iteration cost, while SQUAREM substantially decreases the number of iterations required for convergence. These gains are achieved without compromising numerical stability or likelihood monotonicity.

Overall, the simulation results demonstrate that the proposed Polya-Gamma-based EM algorithm combines stable finite-sample behavior, robustness to approximation choices, and scalability to large datasets. These properties address key practical limitations of existing pogit implementations and support the use of the method in modern large-scale applications.

Finally, to facilitate transparency and reproducibility of the computational evidence reported above, we provide the Julia version 1.12.3 and R version 4.4.2 code used to generate all simulation results in Supplementary File S1.

## 6. Real-World Application

We apply the proposed pogit model, estimated via a Pólya-Gamma-based EM framework, to an openly available dataset derived from Amazon purchase histories [24], with a specific focus on purchases of Apple products within the technology and electronics category. The purpose of this empirical application is to illustrate the use of the model and the practical implementation of the proposed estimation procedure in a realistic, large-scale empirical setting.

This application is not intended to demonstrate superior empirical fit or to provide a comprehensive benchmarking exercise. Rather, it is designed to illustrate the feasibility, interpretability, and numerical stability of the proposed EM framework when applied to a challenging publicly available dataset characterized by severe partial observability and heterogeneous consumer behavior.

The dataset combines detailed longitudinal Amazon purchase histories with respondent-level demographic and household information for N=5027 users in the United States. Data were collected through a consent-centric crowdsourcing protocol, under which participants voluntarily provided exports of their personal Amazon purchase histories spanning 1 January 2018 to 19 March 2023, together with survey-based sociodemographic information. This design yields a rich observational dataset linking transactional behavior over time to individual and household characteristics, while ensuring informed consent and preserving user privacy.

A comprehensive descriptive and exploratory analysis of this dataset has been previously reported in Berke et al. [25]. The dataset and its construction are described in detail in Berke et al. [24] and have since been used in several independent studies examining distinct dimensions of consumer behavior (e.g., [26,27,28]). Building on this established empirical foundation, the present study does not revisit descriptive statistics or exploratory analyses previously reported in the literature, and instead proceeds directly to the model-based analysis under partial observability.

In the present study, the dataset is used under a deliberately constructed information scenario that mirrors the decision environment faced by a focal firm. Although the underlying data contain transactions for multiple brands within the technology and electronics category, the estimation strategy intentionally restricts the information available for model fitting to Apple-specific transactional histories observed on Amazon, together with respondent-level sociodemographic and household characteristics. This restriction is methodological rather than data-driven and reflects the realistic constraint that firms typically lack access to competitors’ sales and to consumers’ total category expenditure. Within this setting of partial observability, the pogit model illustrates how latent wallet allocation and underlying demand intensity can be inferred using focal-firm transaction data alone.

The model specification captures two distinct latent behavioral mechanisms. An intensity component governs the customer’s overall demand for technology and electronics, defined on a normalized latent scale and corresponding to the Size-of-Wallet (SioW). An allocation component governs the fraction of this latent category demand allocated to the focal firm, corresponding to the share-of-wallet (SoW). Identification under partial observability is achieved through the joint hierarchical structure of the model and a deliberate separation of covariates across these latent components. Apple-specific transactional and behavioral features enter the allocation mechanism, whereas broader sociodemographic and household characteristics enter the intensity mechanism.

In this empirical application, all customers are observed over the same calendar window and are, therefore, assumed to face identical exposure. Accordingly, the offset term is fixed at a constant value, $E_{i}\equiv 1$ for all *i*, and cross-sectional heterogeneity in overall purchasing activity is captured entirely through the latent intensity $\lambda_{i}=\exp\;\left(x_{2i}^{\prime }\beta_{2}\right)$.

To operationalize the model in a count-data setting, we transform Apple expenditure observed on Amazon during the evaluation window $T_{2}$ (1 November 2021 to 19 March 2023) using a variance-to-mean scaling motivated by the moment structure of the Poisson distribution; a model-based and data-driven justification of this transformation is provided in Appendix D. Specifically, total monetary spending is rescaled by a constant *c*, estimated exclusively from the training sample, and subsequently rounded to obtain a count-like response variable compatible with the pogit specification. This transformation induces a normalized discrete scale shared by the observed response $y_{i}$ and the latent exposure $n_{i}$, thereby allowing monetary purchase volumes to be modeled coherently within a Poisson–binomial structure. Importantly, neither $y_{i}$ nor $n_{i}$ should be interpreted as literal counts of physical transactions, orders, or items; both quantities represent discretized proxies for relative spending intensity defined on a common latent scale.

Predictors are computed exclusively from transactions in $T_{1}$ (1 January 2018 to 31 October 2021), whereas the response is computed exclusively from transactions in $T_{2}$ (1 November 2021 to 19 March 2023); the train/test partition is defined at the respondent level and applied consistently across both windows. Categorical predictors encoded as single-selection factors are included using an omitted reference level, which defines the reference group, whereas for multiple-selection categorical variables, all indicator categories are retained. All quantitative covariates are standardized prior to estimation. For quantitative transactional predictors, correlation-based screening is performed using Spearman rank correlations to accommodate potential nonlinearity and heavy-tailed distributions. All data-adaptive preprocessing steps—including winsorization thresholds, standardization parameters, and correlation-screening rules—are learned exclusively on the training sample ($n_{train}=1336$) and subsequently applied unchanged to the held-out test sample ($n_{test}=334$), thereby ensuring strict prevention of information leakage.

Although the full Amazon dataset permits observation of technology and electronics spending across multiple brands within the Amazon channel during the evaluation window $T_{2}$, information on non-Apple spending is used exclusively ex post as a channel-restricted benchmark for validation and contextualization of the model-implied SoW.

To ensure transparency and reproducibility of the empirical application, we provide a complete technical report in Supplementary File S2, including data pre-processing procedures, design matrices, coefficient estimates, and detailed catalogs of predictor and response variables. Reproducible implementations of the real-data application in Julia and R are provided in Supplementary File S3.

Against this methodological background, Table 1 and Table 2 summarize the main empirical patterns recovered by the pogit model under the full specification and after covariate selection, respectively. The full specification incorporates the complete set of covariates considered in the empirical analysis and reveals that most of the model’s explanatory content is concentrated in a relatively small subset of predictors related to Apple-specific transactional behavior and household characteristics—most notably RFM variables, income categories, and platform usage intensity—while the remaining covariates contribute limited additional explanatory power, motivating a more parsimonious specification to facilitate interpretation.

In the full specification (Table 1), variation in SoW is primarily driven by Apple-specific transactional behavior. Both Frequency and Monetary enter with positive and statistically significant coefficients (estimates 0.622 and 0.347, respectively), indicating that customers who purchase Apple products more frequently and spend more on the focal brand allocate a larger share of their latent category demand to Apple, whereas Recency does not reach statistical significance once the full set of transactional and demographic controls is included. Heterogeneity in SioW is mainly associated with household income and platform usage intensity: the Income 100–149k category exhibits a positive and statistically significant association with latent purchase intensity (estimate 0.357), while the highest income group does not reach conventional significance levels. Platform engagement plays a central role, with accounts shared by three individuals (Use = 3) displaying substantially higher latent category demand (estimate 0.589). In addition, the life-event indicator Life: Became pregnant enters with a negative and statistically significant coefficient, suggesting a temporary reduction in latent purchasing intensity, whereas other demographic and life-event controls do not exhibit statistically significant effects.

In the parsimonious specification (Table 2), SoW is sharply characterized by Apple-specific behavioral variables in the allocation component. Frequency and Monetary remain the dominant determinants, with positive and statistically significant coefficients (estimates 0.305 and 0.210, respectively), confirming the central role of repeated purchasing and cumulative spending in shaping wallet allocation toward the focal firm. In contrast to the full model, Recency now enters with a negative and statistically significant coefficient (estimate −0.143), indicating that more recent Apple purchases are associated with a higher share of wallet once irrelevant covariates are removed. Under the same parsimonious specification, heterogeneity in SioW is primarily driven by household income and platform usage intensity: the Income 50–74 k and Income 100–149 k categories are positively and significantly associated with latent category demand (estimates 0.396 and 0.432, respectively), while platform engagement remains a dominant factor, with accounts shared by three individuals (Use = 3) exhibiting a strong and highly significant positive association with intensity (estimate 0.615). Consistent with the full specification, the life-event indicator Life: Became pregnant retains a negative and statistically significant effect, whereas other retained income and usage categories do not reach conventional significance levels.

## 7. Conclusions

This paper develops a scalable expectation-maximization algorithm for the Poisson-logistic (pogit) model, a classical framework for count data subject to partial observability. By exploiting a Polya-Gamma data augmentation, the proposed approach yields a quadratic complete-data log-likelihood and closed-form updates in both the E- and M-steps. As a result, each EM iteration is computationally inexpensive, numerically stable, and well-suited to large datasets.

The primary contribution is methodological. Unlike existing frequentist and Bayesian approaches, which typically rely on generic numerical optimization or Markov chain Monte Carlo methods, the proposed EM formulation scales naturally with sample size and covariate dimension. The algorithm admits deterministic full-batch updates, mini-batch variants based on Robbins–Monro stochastic approximation, and quasi-Newton acceleration via SQUAREM, all targeting the same observed-data likelihood. Simulation experiments confirm stable finite-sample behavior, robustness to the negative-binomial approximation underlying the Polya-Gamma construction, and substantial computational gains relative to direct maximum-likelihood optimization.

The proposed framework also suggests several directions for future research. First, the quadratic structure of the M-step makes the algorithm particularly amenable to regularization in high-dimensional settings. Penalized extensions based on $\ell_{1}$ penalties or global–local shrinkage priors, such as the horseshoe, could be incorporated either through penalized M-steps or additional latent-variable augmentations. Second, the Polya-Gamma construction naturally links the present EM approach to variational Bayes methods. In a related direction, exploring variational Bayes approximations built on similar augmentation schemes may yield fast approximate Bayesian procedures that complement the EM algorithm developed here.

More broadly, the results highlight the role of Polya-Gamma augmentation as a unifying computational device for efficient inference in models combining nonlinear link functions and discrete outcomes. By making frequentist estimation of pogit models feasible at scale, the proposed EM algorithm expands the practical applicability of these models in modern empirical settings.

Finally, it is worth noting that the proposed EM estimator targets the maximum–likelihood solution of the pogit model, and therefore, inherits the usual asymptotic properties of likelihood–based inference under standard regularity conditions. While Bayesian Polya-Gamma formulations naturally provide finite-sample posterior uncertainty, the present approach relies on asymptotic inference based on the observed Fisher information, yielding a simple and scalable frequentist alternative suited to large datasets.

## Figures and Tables

**Table 1:** Estimated coefficients for the pogit model (full specification). Confidence intervals are at the 95% level. Statistically significant *p*-values (5%) are shown in bold.

Covariate	Estimates	*p*-Value ^*g*,*h*^	Lower CI	Upper CI
**SoW component**				
Intercept	−0.736	0.044	−1.452	−0.021
Recency	−0.155	0.207	−0.396	0.086
Frequency	0.622	<0.001	0.289	0.956
Monetary	0.347	0.004	0.111	0.582
A.M.Spend a	0.158	0.834	−1.320	1.637
M.M.Spend b	−0.073	0.919	−1.483	1.337
R.A.M.Units c	−0.026	0.866	−0.333	0.280
**SioW/intensity component**				
Intercept	−0.437	0.207	−1.115	0.241
Race: Black d	0.345	0.166	−0.144	0.833
Race: White d	0.262	0.262	−0.196	0.721
Race: Asian d	0.330	0.168	−0.139	0.800
Race: Other d	0.234	0.479	−0.414	0.882
Race: Native American d	−0.424	0.344	−1.303	0.454
Race: Pacific Islander d	−0.606	0.515	−2.431	1.220
Life: Lost job d	−0.283	0.093	−0.613	0.047
Life: Moved residence d	−0.104	0.379	−0.335	0.128
Life: Divorce d	0.132	0.792	−0.849	1.113
Life: Had a child d	0.003	0.991	−0.523	0.529
Life: Became pregnant d	−0.863	0.027	−1.630	−0.096
Age 25–34 ^*e*^	0.027	0.851	−0.255	0.309
Age 35–44 ^*e*^	−0.032	0.843	−0.345	0.281
Age 45–54 ^*e*^	0.169	0.309	−0.156	0.493
Age 55–64 ^*e*^	0.263	0.183	−0.124	0.649
Age ≥ 65 ^*e*^	−0.499	0.153	−1.183	0.186
Income 50–74 k ^*e*^	0.326	0.035	0.022	0.630
Income 75–99 k ^*e*^	0.097	0.572	−0.239	0.433
Income 100–149 k ^*e*^	0.357	0.021	0.053	0.661
Income ≥ 150 k ^*e*^	0.301	0.070	−0.025	0.626
Income < 25 k ^*e*^	−0.016	0.941	−0.438	0.406
Income: Prefer not to say ^*e*^	−0.021	0.963	−0.909	0.867
Use = 2 ^*e*^	−0.023	0.840	−0.247	0.201
Use = 3 ^*e*^	0.589	<0.001	0.323	0.855
Use = 4+ ^*e*^	0.281	0.131	−0.084	0.646
States f	0.014	0.748	−0.069	0.096

^*a*^ A.M.Spend: average monthly spend (USD) over the last 12 months. ^*b*^ M.M.Spend: maximum monthly spend (USD) over the last 12 months. ^*c*^ R.A.M.Units: ratio of average monthly units to peak monthly units (12-month window). ^*d*^ Race, Life: multiple-selection indicator variables. ^*e*^ Age, Income, Use: single-selection factor indicators. ^*f*^ States: number of distinct shipping states. ^*g*^ The *p*-values were computed assuming normality. ^*h*^ The underlying variances were computed with the open product gradient method.

**Table 2:** Estimated coefficients for the pogit model after covariate selection. Confidence intervals are at the 95% level. Statistically significant *p*-values (5%) are shown in bold.

Covariate	Estimates	*p* -Value c,d	Lower CI	Upper CI
**SoW component**				
Intercept	−2.390	0.123	−5.429	0.650
Recency	−0.143	0.022	−0.265	−0.020
Frequency	0.305	0.002	0.114	0.497
Monetary	0.210	0.002	0.078	0.341
**SioW/intensity component**				
Intercept	1.078	0.445	−1.691	3.848
Life: Lost job a	−0.122	0.438	−0.431	0.187
Life: Moved residence a	−0.136	0.238	−0.362	0.090
Life: Divorce a	0.088	0.860	−0.885	1.060
Life: Had a child a	0.024	0.925	−0.483	0.531
Life: Became pregnant a	−0.858	0.026	−1.610	−0.105
Income 50–74 k ^*b*^	0.396	0.010	0.093	0.700
Income 75–99 k ^*b*^	0.236	0.160	−0.093	0.565
Income 100–149 k ^*b*^	0.432	0.005	0.132	0.733
Income ≥ 150 k ^*b*^	0.318	0.059	−0.012	0.648
Income < 25 k ^*b*^	0.068	0.750	−0.350	0.486
Income: Prefer not to say b	0.170	0.693	−0.674	1.014
Use = 2 b	0.032	0.776	−0.186	0.250
Use = 3 b	0.615	<0.001	0.354	0.875
Use = ${\mathrm{4+}}^{b}$	0.277	0.126	−0.078	0.633

^*a*^ Life: multiple-selection indicator variables. ^*b*^ Income, Use: single-selection factor indicators. ^*c*^ The *p*-values were computed assuming normality. ^*d*^ The underlying variances were computed with the open product gradient method.

**Figure A1:**
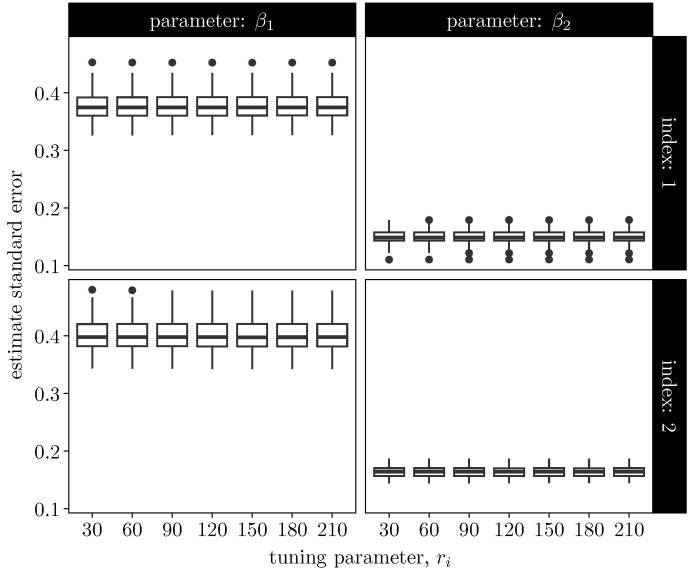
Estimate standard errors as a function of the tuning parameter $r_{i}$. The standard errors are relatively stable across values of $r_{i}$.

**Figure A2:**
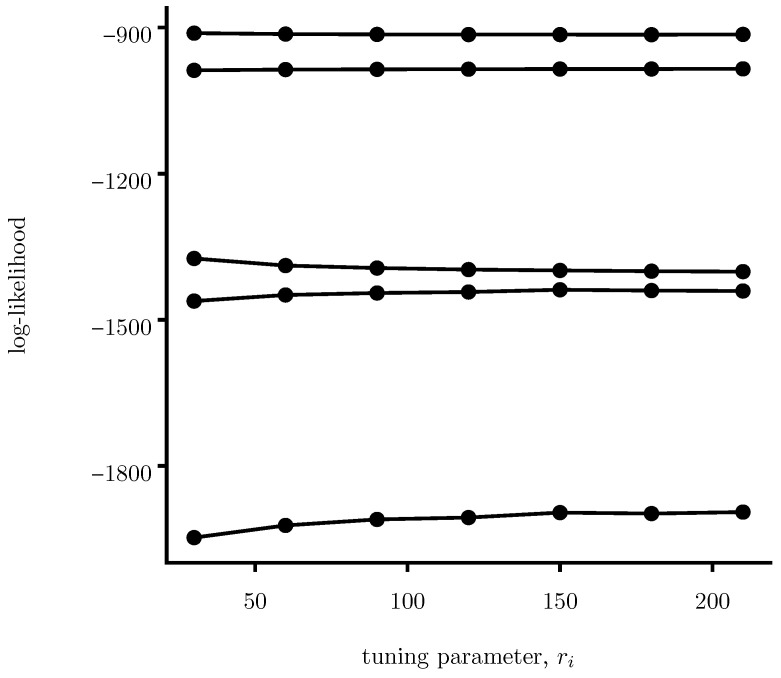
Observed log-likelihood as a function of the tuning parameter $r_{i}$, for 5 simulated datasets. The log-likelihood are relatively stable for $r_{i}\geq 100$.

**Figure A3:**
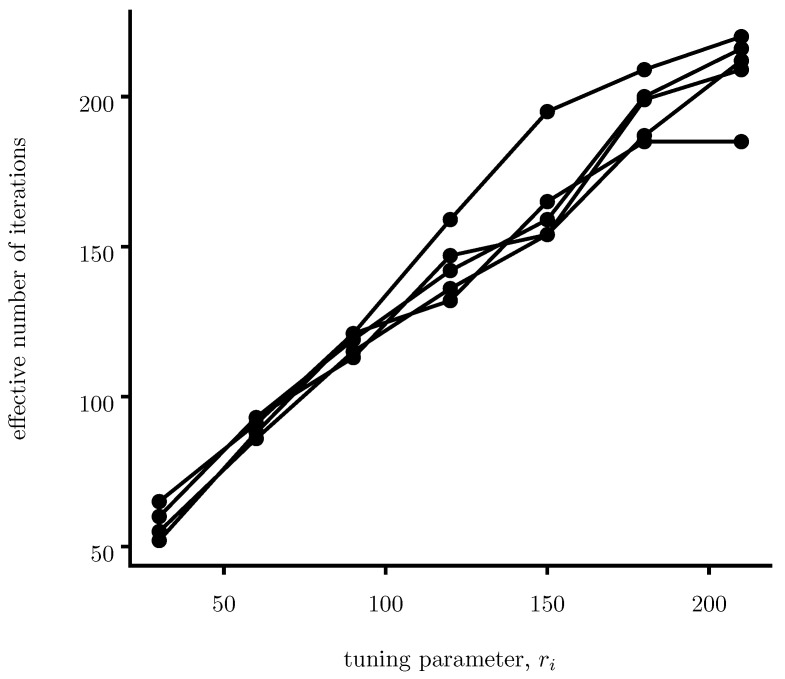
Effective number of iterations as a function of the tuning parameter $r_{i}$, for 5 simulated datasets. The log-likelihood are relatively stable for $r_{i}\geq 100$.

## Data Availability

The empirical application in our manuscript is based on an open-access dataset, originally introduced and documented in Berke et al. [24]. No proprietary or restricted data are used in this study. While the raw data are openly available from the original source, the variables analyzed in our paper are derived and constructed by the authors through preprocessing and feature-engineering steps required for the implementation of the proposed pogit model. The original contributions presented in this study are included in the article. Further inquiries can be directed to the corresponding authors.

## Supplementary Materials

The following supporting information can be downloaded at: https://www.mdpi.com/article/10.3390/e28020207/s1. Supplementary File S1: Replication package for the simulation experiments (scripts to reproduce the simulation study) (ZIP). Supplementary File S2: Report of the real application (variable catalog, design-matrix construction, and preprocessing details) (PDF). Supplementary File S3: Replication package for the real application (datasets and Julia/R scripts to construct the design matrices and reproduce the empirical analyses) (ZIP). Reference [29] is cited in the Supplementary Materials.

## Appendix A. Local Identification Conditions

In this section, we study the local identification of the pogit parameters. Local identification is the relevant notion in our context, as it characterizes whether parameters can be uniquely recovered—up to arbitrarily small perturbations—from the distribution of the observed data, and it is the standard concept underlying asymptotic inference in nonlinear parametric models.

We write $x_{i}^{\prime }=\left(x_{i1}^{\prime },x_{i2}^{\prime }\right)$, where $x_{i1}$ and $x_{i2}$ denote the covariate vectors associated with the reporting and intensity components, respectively.

Throughout this section, $x_{i}$ denotes the vector of observed covariates (regressors), while $y_{i}$ denotes the observed count response.

**Definition A1.** 

*Let $\theta \in \Theta \subset R^{M}(M<\infty )$ denote the vector of model parameters, and let $P_{\theta }$ denote the probability distribution of the observed data $x=(x_{1},\ldots ,x_{n})$. Two parameter points θ and $\theta^{\prime }$ are said to be observationally equivalent if $P_{\theta }=P_{\theta^{\prime }}$. A parameter point $\theta_{0}$ is locally identified if there exists an open neighborhood $U(\theta_{0})$ such that no $\theta \in U\left(\theta_{0}\right)\setminus \left\{\theta_{0}\right\}$ is observationally equivalent to $\theta_{0}$.*

Local identification ensures that the likelihood function has a unique maximizer in a neighborhood of the true parameter value, ruling out local ridges or flat directions that would prevent reliable inference.

Rothenberg [30] provides sufficient conditions for local identification. In order to express them succinctly, we need the notions of regular models and regular points.

**Definition A2.** 

*Consider a parametric statistical model (S,F), where S is the sample space and F={f(·∣θ):θ∈Θ} is a family of probability densities or mass functions. The model is said to be regular if*

1. *Θ* *is an open subset of $R^{M}$, for some M<∞;*
2. *the support of f(·∣θ) does not depend on θ;*
3. *there exists an open set Ψ⊇Θ such that f(y∣θ) and logf(y∣θ) are continuously differentiable with respect to θ for all θ∈Ψ and all y in the support;*
4. *the Fisher information matrix*
  $$I\left(\theta \right)=E\;\left[\nabla_{\theta }\log f\left(y|\theta \right)\nabla_{\theta }\log f{\left(y|\theta \right)}^{\prime }\right]$$
  *exists and is continuous on Θ.*

**Definition A3.** 
*Let M(θ) be a matrix whose entries are continuous functions of θ on *Θ*. A point $\theta_{0}\in \Theta$ is called a regular point of M(·) if there exists an open neighborhood of $\theta_{0}$ over which M(θ) has constant rank.*

**Remark A1.** 

*These definitions extend immediately to regression models by replacing logf(y∣θ) with logf(y∣x,θ) and taking expectations over the joint distribution of (x,y).*

Under these conditions, local identification can be characterized entirely in terms of the Fisher information matrix.

**Theorem A1** 
(Rothenberg, 1971)**.** *Consider a regular parametric model with parameter θ∈Θ and Fisher information matrix I(θ). If $\theta_{0}$ is a regular point of I(θ), then $\theta_{0}$ is locally identified if and only if $I(\theta_{0})$ is non-singular.*

With these definitions and theorems, we are now ready to prove Theorem 1. To this end, we start with the following lemma:

**Lemma A1.** 

*Given a pogit model, define $z_{i}^{\prime }=\left(\left(1-\theta_{i}\right)x_{i1}^{\prime },x_{i2}^{\prime }\right)$. Suppose $\left\{\left(y_{i},x_{i}\right)\right\}$ is an i.i.d. sequence. Then, the Fisher information is given by $I\left(\beta \right)=NI_{1}\left(\beta \right)$, with $I_{1}\left(\beta \right)=E\left[\theta_{1}\lambda_{1}z_{1}z_{1}^{\prime }\right]$.*

**Proof.** For i.i.d. data, the Fisher information is $I\left(\beta \right)=NE[E\left[s_{1}s_{1}^{\prime }|x\right]]$, where $s_{1}$ is the 1st contribution to the score function. To compute such a contribution, observe that the first contribution to the likelihood is

$$\begin{array}{c} \ell_{1}=\log\mathrm{Poisson}\left(y_{1}|\lambda_{1}\theta_{1}\right)=-\theta_{1}\lambda_{1}+y_{1}\log\left(\theta_{1}\lambda_{1}\right)-\log y_{1}!, \end{array}$$

Deriving this expression with respect to $\beta_{1}$ and $\beta_{2}$, the following is obtained

$$\begin{array}{c} s_{1}\left(\beta \right)=\left[\begin{array}{c} \nabla_{\beta_{1}}\ell_{1}\\ \nabla_{\beta_{2}}\ell_{1} \end{array}\right]=\left[\begin{array}{c} \left(y_{1}-\theta_{1}\lambda_{1}\right)\left(1-\theta_{1}\right)x_{11}\\ \left(y_{1}-\theta_{1}\lambda_{1}\right)x_{12} \end{array}\right]=\left(y_{1}-\theta_{1}\lambda_{1}\right)z_{1}. \end{array}$$

Thus, $s_{1}s_{1}^{\prime }={\left(y_{1}-\theta_{1}\lambda_{1}\right)}^{2}z_{1}z_{1}^{\prime }$. Now, under the model, $\left(y_{1}|x_{1}\right)∼\mathrm{Poisson}\left(\lambda_{1}\theta_{1}\right)$. Hence, exploiting some well-known properties of the Poisson distribution, the following expression is obtained

$$\begin{array}{c} E\left[{\left(y_{1}-\theta_{1}\lambda_{1}\right)}^{2}|x_{1}\right]=V\left[y_{1}|x_{1}\right]=\theta_{1}\lambda_{1}, \end{array}$$

so $E\left[s_{1}s_{1}^{\prime }|x_{1}\right]=\theta_{1}\lambda_{1}z_{1}z_{1}^{\prime }$ and the result follows. □

With this result, we can now prove Theorem 1.

**Proof of Theorem 1.** Clearly, the pogit model is regular, so it only remains to prove that $I(\beta_{0})$ is non-singular for any regular point $\beta_{0}$. On the other hand, by Lemma A1, $I\left(\beta_{0}\right)=NE\left[\theta_{1}\lambda_{1}z_{1}z_{1}^{\prime }\right]$ (with β evaluated at $\beta_{0}$). Hence, it suffices to prove that $E[\theta_{1}\lambda_{1}z_{1}z_{1}^{\prime }]$ is a positive definite matrix. To do so, suppose the opposite. Then,

$$\begin{array}{c} \xi^{\prime }E\left[\theta_{1}\lambda_{1}z_{1}z_{1}^{\prime }\right]\xi =E\left[\theta_{1}\lambda_{1}{\left(z_{1}^{\prime }\xi \right)}^{2}\right]=0 \end{array}$$

for some $\xi \neq 0_{K_{1}+K_{2}}$, where $K_{1}$ and $K_{2}$ denote the dimensions of $x_{i1}$ and $x_{i2}$, respectively, which implies that $z_{i}^{\prime }\xi =0$ (a.s.) because $\theta_{1}\lambda_{1}>0$, but $z_{1}^{\prime }\xi$ cannot be zero (a.s.) because then $E[z_{1}z_{1}^{\prime }]=0$, so $P(z_{1}^{\prime }\xi =0)<1$; yielding a contradiction. □

## Appendix B. Full Derivation of the Complete Likelihood

Throughout this section, for each observational unit i=1,…,N, we work with linear predictors $\eta_{i1}=x_{i1}^{\prime }\beta_{1}$ and $\eta_{i2}=x_{i2}^{\prime }\beta_{2}$, which correspond to the binomial (reporting) and latent intensity components of the pogit model, respectively. For notational convenience, likelihood expressions are often written in terms of these linear predictors rather than the natural parameters $\theta_{i}$ and $\lambda_{i}$, where $\theta_{i}=\mathrm{sigmoid}\left(\eta_{i1}\right)$ and $\lambda_{i}=\exp\left(\eta_{i2}\right)$.

Before proceeding, note that the auxiliary quantities *a*, *b*, and ψ introduced below are temporary variables used solely to apply the Polya-Gamma identity in Property 2. They are explicitly defined within each likelihood component and should not be interpreted as structural parameters of the pogit model.

Throughout the augmented likelihood derivations, $E_{w_{ij}}$ denotes expectation with respect to the Polya-Gamma latent variable $w_{ij}$, with j∈{1,2}, introduced by Property 2.

Here $r_{i}\gg 0$ is a fixed approximation parameter controlling the negative-binomial approximation to the Poisson distribution.

### Appendix B.1. Binomial Component

We know $\theta_{i}=\mathrm{sigmoid}\left(\eta_{i1}\right)$. Hence,

$$\begin{array}{cc} \mathrm{Bin}(y_{i}|n_{i},\theta_{i}) & \propto \theta_{i}^{y_{i}}{\left(1-\theta_{i}\right)}^{n_{i}-y_{i}}\\ \; & \propto \exp{\left(\eta_{i1}\right)}^{y_{i}}/{\left(1+\exp\left(\eta_{i1}\right)\right)}^{n_{i}}\\ \; & \propto {\left(e^{\psi }\right)}^{a}/{\left(1+e^{\psi }\right)}^{b}, \end{array}$$

where $\psi =\eta_{i1}$, $a=y_{i}$ and $b=n_{i}$. Applying Property 2 gives the desired result.

### Appendix B.2. Poisson Component

We know $\lambda_{i}=e^{\eta_{i2}}$ so $E_{i}\lambda_{i}=r_{i}e^{\eta_{i2}-\log\left(r_{i}/E_{i}\right)}$ and

Poisson(ni∣Eiλi)≈NegBin(ni∣ri,Eiλi/(ri+Eiλi))∝ri+ni−1ri−1riri+EiλiriEiλiri+Eiλini∝(rieηi2−log(ri/Ei))ni(ri+rieηi2−log(ri/Ei))ni+ri∝(eηi2−log(ri/Ei))ni(1+eηi2−log(ri/Ei))ni+ri∝(eψ)a/(1+eψ)b

where $\psi =\eta_{i2}-\log\left(r_{i}/E_{i}\right)$, $a=n_{i}$ and $b=r_{i}+n_{i}$. Applying Property 2 gives

$$\begin{array}{c} \mathrm{Poisson}(n_{i}|E_{i}\lambda_{i})\propto E_{w_{i2}}\exp\left\{\left(a-b/2\right)\psi -w_{i2}\psi^{2}/2\right\} \end{array}$$

with $w_{i2}∼\mathrm{PG}\left(b,0\right)$. However,

$$\begin{array}{cc} (a-b/2)\psi & ≐\left(a-b/2\right)\eta_{i2},\\ w_{i2}\psi^{2}/2 & ≐w_{i2}\eta_{i2}^{2}/2-w_{i2}\log\left(r_{i}/E_{i}\right)\eta_{i2}+\frac{w_{i2}}{2}\;{\left(\log(r_{i}/E_{i})\right)}^{2}. \end{array}$$

Noting that the symbol ≐ denotes equality up to additive terms independent of the linear predictor and absorbable into the normalizing constant, we have

$$\begin{array}{cc} \; & \left(a-b/2\right)\psi -w_{i2}\psi^{2}/2\\ \; & ≐\left(a-b/2\right)\eta_{i2}-w_{i2}\eta_{i2}^{2}/2+w_{i2}\log\left(r_{i}/E_{i}\right)\eta_{i2}-\frac{w_{i2}}{2}{\left(\log(r_{i}/E_{i})\right)}^{2}\\ \; & ≐\left(\left(r_{i}-n_{i}\right)/2+w_{i2}\log\left(r_{i}/E_{i}\right)\right)\eta_{i2}-w_{i2}\eta_{i2}^{2}/2-\frac{w_{i2}}{2}{\left(\log(r_{i}/E_{i})\right)}^{2}\\ \; & ≐\kappa_{i2}\eta_{i2}-w_{i2}\eta_{i2}^{2}/2-\frac{w_{i2}}{2}{\left(\log(r_{i}/E_{i})\right)}^{2}, \end{array}$$

with $\kappa_{i2}$ defined in the main document.

Exponentiating and taking expectations, we obtain

$$\begin{array}{c} \mathrm{Poisson}(n_{i}|E_{i}\lambda_{i})\propto E_{w_{i2}}\exp\{\kappa_{i2}\eta_{i2}-w_{i2}\eta_{i2}^{2}/2-\frac{w_{i2}}{2}{\left(\log(r_{i}/E_{i})\right)}^{2}\}, \end{array}$$

as stated in the main document.

## Appendix C. Additional Simulation Experiments

This appendix reports additional simulation experiments designed to assess the impact of the Poisson-to-negative binomial approximation underlying the Polya–Gamma augmentation on likelihood–based inference. In particular, we examine the sensitivity of the standard errors, effective iterations and observed–data likelihood to the choice of the approximation parameter. These supplementary results complement the main simulation study by providing direct diagnostics for inferential stability and support the practical recommendations adopted in the main text.

Figure A1 illustrates how likelihood–based standard errors vary with the negative–binomial approximation parameter *r*, where the standard errors where calculated using OPG (outer product gradient) method. To this end, we simulated 100 datasets of size N=500 using the same data-generating process used in Section 5. Across the range of values considered, the estimated standard errors exhibit only minor fluctuations and no discernible trend as *r* increases. This indicates that, in the simulation settings examined, inferential uncertainty is largely insensitive to the precise choice of *r* once it is sufficiently large.

Figure A2 shows how the observed-data log-likelihood varies with the negative-binomial approximation parameter *r*. To improve readability and avoid an overcrowded spaghetti plot, we track the likelihood trajectories for five simulated datasets of size N=500, generated as described in Section 5. As shown in the figure, the likelihood increases rapidly for small values of *r* and then stabilizes as *r* grows. In all trajectories, changes become negligible once *r* reaches values around r≈100, indicating that the approximation is sufficiently accurate beyond this point. This behavior supports our practical recommendation to use moderate values of *r*, as larger choices yield no meaningful improvement in likelihood.

Figure A3 documents how the effective number of EM iterations varies with the approximation parameter *r*. The number of iterations increases monotonically with *r*, indicating higher computational cost for more refined approximations. At the same time, earlier results show that estimates, standard errors, and likelihood values stabilize for moderate values of *r*. Taken together, these findings suggest that choosing *r* around 100 is sufficient in practice, as larger values mainly increase runtime without yielding material inferential gains.

## Appendix D. Discretization of Monetary Spending

Following Glady and Croux [2], when monetary volumes are observed, spending can be discretized into count-like units prior to estimation in Poisson-based models. In the real data application, we consider Apple spending on Amazon within the technology/electronics universe U over the evaluation window $T_{2}$, denoted by ${\mathrm{spend}}_{i}^{\mathrm{Apple}|\mathrm{Amz},U}$.

This appendix provides a methodological justification of the discretization procedure used in the real-world application, grounded in the statistical properties of the data and the Poisson modeling framework. To avoid information leakage, all quantities used to define the discretization scale are computed exclusively on the training sample. Let $I_{\mathrm{tr}}$ denote the training index set. We define the discretization constant as

$$\begin{array}{c} c=\frac{{\hat{\sigma }}_{\mathrm{tr}}^{2\mathrm{Apple}|\mathrm{Amz},U}}{{\bar{\mathrm{spend}}}_{\mathrm{tr}}^{\mathrm{Apple}|\mathrm{Amz},U}}, \end{array}$$

that is, the ratio between the cross-sectional variance and the mean of Apple spending in the training set. This choice is motivated by the Poisson assumption underlying the model, under which the mean and variance coincide on the latent model scale.

Prior to computing *c*, extreme values are mitigated using a Tukey rule-based winsorization procedure, with thresholds estimated on the training sample and subsequently applied unchanged to the test sample. The discretized response is then defined as

$$\begin{array}{c} y_{i}=\mathrm{round}\;\left(\frac{{\mathrm{spend}}_{i}^{\mathrm{Apple}|\mathrm{Amz},U}}{c}\right). \end{array}$$

The operator round(·) denotes rounding to the nearest integer. The resulting variable $y_{i}$ is a normalized, count-like proxy for monetary spending that is compatible with the Poisson likelihood employed in the pogit model. Importantly, neither $y_{i}$ nor the latent category size $n_{i}$ should be interpreted as literal counts of transactions or items; both quantities are defined on a common discretized scale designed to capture relative spending intensity.
