# Supplementary material for: Efficient EM Estimation for the Pogit Model via Polya-Gamma Augmentation
Source: Entropy (Basel). 2026 Feb 11;28(2):207. doi: 10.3390/e28020207 (PMC12939104; doi:10.3390/e28020207)
Supplement: Supplementary file 1 [file Supplementary_File_S2.pdf › Supplementary_File_S2.pdf]

entropy

MDPI

Article

Supplementary File S2: Report of the real application

Iván Gutiérrez [1,*], Sandra Ramírez [2,*] and Leonardo Jofré [3]

[1] Departamento de Economía y Administración, Facultad de Economía y Negocios, Universidad Andrés Bello, 376000 Santiago, Chile
[2] Departamento de Ciencias Naturales y Matemáticas, Facultad de Ingeniería y Ciencias, Pontificia Universidad Javeriana, 760031 Cali, Colombia
[3] Departamento de Estadística, Facultad de Matemáticas, Pontificia Universidad Católica de Chile, 7820436 Santiago, Chile; lnjofre@mat.uc.cl
* Correspondence: ivan.gutierrez@unab.cl (I.G.); smramirez@javerianacali.edu.co (S.R.)

Abstract

This appendix documents the empirical implementation of the *pogit* model proposed in the main body of the paper, applied to real transaction data from Amazon. Its purpose is purely expository: to provide a transparent and self–contained account of the data construction, variable engineering, preprocessing steps, estimation details, and validation procedures underlying the real–world application. No new methodological elements are introduced in this appendix. All modeling assumptions, likelihood specifications, and inferential principles follow directly from the pogit framework developed in the paper and from closely related formulations in the prior literature. The material presented here is intended to facilitate reproducibility, clarify empirical design choices under partial observability, and support the interpretation of the empirical results reported in the main text.

1. Model

The model estimates customers' *Share–of–Wallet* (SoW) for Apple and their *Size–of–Wallet* (SioW) in the technology/electronics category without relying on survey data in which customers self–report wallet shares.

We assume the availability of a sample $\{(y_i, \boldsymbol{x}_{1i}, \boldsymbol{x}_{2i}, E_i)\}_{i=1}^{N}$. In this sample, y_i denotes a discretized, count-like *proxy* constructed from customer i's Apple spending observed on Amazon within the technology/electronics universe over the evaluation window (see Section 5). Importantly, y_i is obtained by rescaling monetary spending by a training-based constant c and rounding; thus, it should be interpreted as a normalized spending proxy rather than as a literal count of transactions or units. The quantity n_i represents the customer's total (latent) technology/electronics purchase volume across all brands and retail channels, expressed on the same normalized (discretized) scale as y_i.

Although the underlying Amazon transaction logs contain purchases from multiple brands, only Apple transactions are used to construct transaction–derived predictors. This design choice explicitly mimics the informational environment of a focal firm, which typically observes its own customer–brand interactions but not competitors' sales or off–platform purchases.

The vector $\boldsymbol{x}_{1i} = (x_{1ij}) \in \mathbb{R}^J$ contains the J regressors associated with the *allocation mechanism* governing the Apple SoW of customer i. These covariates consist of Apple–specific transactional summaries and capture variation in brand allocation behavior. Similarly, the vector $\boldsymbol{x}_{2i} = (x_{2i\ell}) \in \mathbb{R}^L$ contains the L regressors associated with the *intensity mechanism*, which governs the customer's overall category demand (SioW).

Check for updates

Academic Editor: Michel Broniatowski

Received: 12 January 2026
Revised: 30 January 2026
Accepted: 5 February 2026
Published:

Copyright: © 2026 by the authors. Licensee MDPI, Basel, Switzerland. This article is an open access article distributed under the terms and conditions of the Creative Commons Attribution (CC BY) license.

The scalar $E_i > 0$ denotes an *offset (exposure)* capturing exogenous differences in purchasing opportunities (e.g., time at risk or market exposure). In the empirical setting considered here, all respondents are observed over the same calendar window and are assumed to face comparable exposure. Accordingly, we set $E_i \equiv 1$ for all i, so that cross-sectional heterogeneity in overall category intensity is modeled entirely through the covariates in $\boldsymbol{x}_{2i}$ and the intercept in the intensity component.

The model is specified hierarchically as

$$
\begin{aligned}
y_i \mid n_i, \theta_i &\sim \text{Bin}(n_i, \theta_i), \qquad i = 1, \ldots, N, \\
n_i \mid \lambda_i &\sim \text{Poisson}(\lambda_i), \\
\log(\lambda_i) &= \log(E_i) + \boldsymbol{x}'_{2i}\boldsymbol{\beta}_2, \\
\theta_i &= \text{sigmoid}(\boldsymbol{x}'_{1i}\boldsymbol{\beta}_1),
\end{aligned}
$$

where $\text{sigmoid}(x) = \{1 + \exp(-x)\}^{-1}$ denotes the sigmoid function.

2. Data

The dataset used in this study derives from a consent-centric crowdsourcing protocol that was reviewed and approved by the MIT Institutional Review Board (IRB; protocol #2205000649) [1]. The authors deployed a custom survey for U.S. consumers that captured respondent-level covariates (e.g., demographics, lifestyle, and health) and offered an optional, secure mechanism for contributing individual Amazon purchase histories.

The released corpus comprises more than 1.8 million transactions from 5,027 U.S. Amazon.com consumers spanning 2018–2023. Core transactional fields include order date, product code, product title, unit price, quantity, and the U.S. state of the shipping address.

Building on this corpus, the sample and modeling windows are constructed as follows. Two non-overlapping horizons are defined: a *feature window* T_1 from 2018-01-01 to 2021-10-31 and a *response/evaluation window* T_2 from 2021-11-01 to 2023-03-19. Using respondent demographics and lifestyle variables from the survey, together with Amazon transactions observed during T_1, the full set of predictor variables is engineered (e.g., recency/frequency/monetary features, seasonal aggregates, category intensities, and purchase-based signals). The response variable used by the models is computed from Amazon transactions in T_2. In addition—because ground-truth, survey-based SoW for Apple is not observed—An Amazon-bounded SoW is computed in T_2 and used exclusively for ex post validation.

To ensure that Apple-related propensity and intensity are well defined, the sample is restricted to respondents with at least one Apple purchase during T_1, yielding a working cohort of $N = 2{,}090$ individuals. This cohort is randomly partitioned into training (80%) and test (20%) sets. The models are fitted on the training set using predictors engineered from T_1 and a response variable computed in T_2, and out-of-sample evaluation is conducted on the held-out test set. For validation within the Amazon-bounded universe, test-set estimates are benchmarked against an empirical estimate of Apple's SoW in T_2, defined for each respondent as Apple spend divided by total technology/electronics spend on Amazon (Apple plus other brands).

3. Construction of Predictor Variables

Raw inputs are consolidated into a customer–level matrix—one row per customer, with columns corresponding to features engineered from transaction records and linked survey covariates. The analytical sample is restricted to customers with at least one Apple purchase in T_1, ensuring that Apple–specific transactional features used in the allocation

component are well defined; customers without Apple purchases in T_1 are excluded from Apple–focused analyses.

Survey responses are one-hot encoded after removing a reference level for each construct to avoid collinearity. Specifically, features #1–#51 comprise binary indicators derived from: age group; Hispanic/Latino origin; race (multi-select); highest educational attainment; household income; gender identity; sexual orientation; U.S. state of residence (50 states, District of Columbia, and Puerto Rico); account-sharing intensity ("How many people do you share your Amazon account with?"); household size; Amazon ordering frequency; substance-use items for cigarettes, marijuana, and alcohol (asked at the respondent/household/account-sharing level); health status (diabetes; wheelchair use); and major life changes during 2021 (multi-select).

Feature #52 is defined as the number of distinct U.S. states from which a customer's orders were fulfilled, computed as the count of unique state codes within T_1.

The next features #53–#77 are computed over the 12-month trailing window ending on 2021-10-31 (i.e., from 2020-11-01 to 2021-10-31). Each feature is provided in two parallel versions: a dollar-valued variant, in which spending is aggregated and reported in USD, and a product-count variant, in which distinct items purchased are counted. In addition, a coverage measure is included, defined as the number of months with at least one purchase in the last twelve months.

The classical RFM (Recency, Frequency, Monetary) framework is employed to explain customer-level heterogeneity in SoW component of the model. Features #78–#80 are brand-specific, RFM-style measures for Apple computed over the full historical interval T_1: frequency is the count of Apple transactions within T_1; monetary is the sum of line-item totals for Apple within T_1, where each line total equals *unit_price* $\times$ *quantity* (net of item-level discounts and excluding taxes and shipping); and recency_days is the number of days from the window end date (2021-10-31) to the customer's most recent Apple transaction dated within T_1.

All variables described in this subsection are constructed exclusively from information observed during the feature window T_1 and constitute the predictor set used by the pogit model. No information from the evaluation window T_2 enters the construction of these predictors. Quantities derived from T_2, including the model response and Amazon-bounded benchmarks used for validation, are defined separately below.

4. Construction of Amazon SoW for Model Validation

Throughout the empirical application, Apple is treated as the focal firm. The model-implied SoW θ_i is defined over the full technology/electronics universe and represents the fraction of customer i's total category demand allocated to Apple across all brands and retail channels. Because only Amazon transactions are employed, this quantity is is assumed not directly observable from the transaction logs. We therefore construct an Amazon-bounded SoW based solely on observed Amazon purchases within the same product universe, which is used exclusively as an empirical reference for ex post comparison and contextualization of model pestimates. This Amazon-specific SoW is not conceptually equivalent to θ_i, does not enter the likelihood, and plays no role in estimation or calibration of the pogit model.

Let $\mathcal{U}$ denote the product universe (*technology/electronics*). For each order line o, the line value total_price$_o$ is given by the product of the net unit price and the purchased quantity, formally, total_price$_o$ = unit_price$_o$ $\times$ quantity$_o$, computed net of item-level discounts.

The model–implied, cross–channel SoW is $\theta_i \in [0, 1]$, which represents the fraction of customer i's total technology/electronics demand allocated to Apple across all channels during T_2. In the empirical setting, this quantity is treated as latent and is inferred from the pogit model rather than directly observed from transaction logs.

Within the same product universe $\mathcal{U}$ and window T_2, let Amz denote the Amazon channel and $\mathcal{O}_i^{\text{Amz}}(T_2,\mathcal{U})$ the set of order lines for customer i in $\mathcal{U}$ placed on Amazon during T_2. Let $\mathbf{1}\{\text{Apple}(o)\}$ indicate that line o is an Apple product. Define

$$\text{spend}_i^{\text{Apple}|\text{Amz},\mathcal{U}} = \sum_{o\in\mathcal{O}_i^{\text{Amz}}(T_2,\mathcal{U})} \text{total\_price}_o\, \mathbf{1}\{\text{Apple}(o)\},$$
$$\text{spend}_i^{\text{Amz},\mathcal{U}} = \sum_{o\in\mathcal{O}_i^{\text{Amz}}(T_2,\mathcal{U})} \text{total\_price}_o.$$

The *SoW of Apple's customer on Amazon* is defined as

$$\text{SoW}_i^{\text{Apple}|\text{Amz},\mathcal{U},T_2} = \frac{\text{spend}_i^{\text{Apple}|\text{Amz},\mathcal{U}}}{\text{spend}_i^{\text{Amz},\mathcal{U}}} \in [0,1],$$

which measures, for a given customer and period, the proportion of Amazon spending in the technology/electronics universe allocated to Apple products.

Importantly, the Amazon–bounded SoW constructed in this section is used exclusively as an *ex post* reference for validation and visualization and does not enter the likelihood or influence parameter estimation.

5. Construction of Response Variable

Following Glady and Croux [2], when monetary volumes are observed, spending can be discretized into count-like units prior to estimation. In this application, we use Apple spending on Amazon within the technology/electronics universe $\mathcal{U}$ over the evaluation window T_2, denoted by $\text{spend}_i^{\text{Apple}|\text{Amz},\mathcal{U}}$.

To prevent information leakage, all quantities required to determine the discretization scale are computed using training customers only. Let $\mathcal{I}_{\text{tr}}$ denote the index set of training customers, with $N_{\text{tr}} = |\mathcal{I}_{\text{tr}}|$. The training-set mean and variance of Apple spending are defined as

$$\overline{\text{spend}}_{\text{tr}}^{\text{Apple}|\text{Amz},\mathcal{U}} = \frac{1}{N_{\text{tr}}} \sum_{j\in\mathcal{I}_{\text{tr}}} \text{spend}_j^{\text{Apple}|\text{Amz},\mathcal{U}},$$
$$\widehat{\sigma}_{\text{tr}}^{2\,\text{Apple}|\text{Amz},\mathcal{U}} = \frac{1}{N_{\text{tr}}-1} \sum_{j\in\mathcal{I}_{\text{tr}}} \left(\text{spend}_j^{\text{Apple}|\text{Amz},\mathcal{U}} - \overline{\text{spend}}_{\text{tr}}^{\text{Apple}|\text{Amz},\mathcal{U}}\right)^2.$$

Unlike Glady and Croux [2], we set the discretization constant to the ratio between the cross-sectional variance and mean,

$$c = \frac{\widehat{\sigma}_{\text{tr}}^{2\,\text{Apple}|\text{Amz},\mathcal{U}}}{\overline{\text{spend}}_{\text{tr}}^{\text{Apple}|\text{Amz},\mathcal{U}}},$$

which is motivated by the Poisson assumption that the mean and variance coincide on the model scale.

Prior to computing c, extreme values in Apple spending over T_2 are mitigated in two steps: first, a small number of severely anomalous cases are removed using an application–defined extreme Tukey rule ($k = 5$); second, the upper tail is winsorized using a Tukey rule with $k = 3$. To prevent information leakage, both the removal and capping thresholds are estimated on the training set only and then applied unchanged to the test set. The discretization constant c is computed exclusively from the resulting (filtered and winsorized) training spending and held fixed thereafter.

The response variable is defined as

$$y_i = \mathrm{round}\left(\frac{\mathrm{spend}_i^{\mathrm{Apple}|\mathrm{Amz},\mathcal{U}}}{c}\right),$$

and should be interpreted as a normalized (discretized) spending proxy rather than as a literal count of units, orders, or transactions.

For descriptive benchmarking within Amazon, we also construct an empirical Amazon-bounded proxy for SioW,

$$n_i^{\mathrm{emp}} = \mathrm{round}\left(\frac{\mathrm{spend}_i^{\mathrm{Amz},\mathcal{U}}}{c}\right),$$

where $\mathrm{spend}_i^{\mathrm{Amz},\mathcal{U}}$ denotes total technology/electronics spending on Amazon during T_2. This benchmark is used exclusively for ex post comparison and is not part of the pogit likelihood or estimation.

A complete catalog of computed variables is provided in Sections 10.1 and 10.2.

6. Pre-processing

To prevent information leakage, all data-adaptive preprocessing steps are learned using training customers only and then applied unchanged to the held-out test set. Customers are split into training and test sets at the customer-ID level.

Extreme observations in *all quantitative variables* are handled in two stages. First, a small number of severely anomalous cases are removed using an application-defined extreme Tukey rule ($k = 5$). Second, upper-tail winsorization is applied using Tukey caps with $k = 3$. In both stages, all thresholds are estimated on the training split and then reused unchanged for the test split. Binary indicators are unaffected by construction.

After outlier handling, continuous covariates are standardized using training-set means and standard deviations, and the same centering and scaling parameters are applied to the test set. When redundancy control is required among quantitative transactional covariates, correlation-based screening is performed on the training split only (using Spearman correlations with a fixed cutoff), and the selected set is carried forward unchanged.

Single-choice categorical covariates are dummy encoded with one reference level omitted per factor to ensure identifiability. Multi-select constructs (e.g., life events, race) are encoded as a full set of binary indicators, since categories are not mutually exclusive.

7. Estimated Coefficients for the Allocation (SoW) and Intensity (SioW) Components

This subsection reports and interprets the estimated coefficients associated with the two latent mechanisms of the pogit model in the Amazon application: (i) the *allocation* component governing SoW, and (ii) the *intensity* component governing SioW. These two components represent distinct latent processes: the former captures the probability that category demand is allocated to the focal brand (Apple), while the latter captures the overall intensity of category consumption.

The allocation mechanism is specified as

$$\theta_i = \mathrm{sigmoid}\big(\boldsymbol{x}_{1i}'\boldsymbol{\beta}_1\big),$$

so that the coefficients $\boldsymbol{\beta}_1$ act on the *log-odds* of allocating category demand to Apple. Positive coefficients increase the probability of allocation, whereas negative coefficients reduce it, ceteris paribus.

The intensity mechanism is specified as

$$\log(\lambda_i) = \log(E_i) + \boldsymbol{x}_{2i}'\boldsymbol{\beta}_2,$$

so that the coefficients $\boldsymbol{\beta}_2$ act *log-linearly* on the latent category intensity λ_i. In the present application, exposure is normalized to $E_i \equiv 1$, so that λ_i depends exclusively on the covariates $\boldsymbol{x}_{2i}$.

Let $\hat{\boldsymbol{\beta}}_1$ and $\hat{\boldsymbol{\beta}}_2$ denote the maximum-likelihood estimates of the allocation and intensity parameters, respectively. Conditional on these estimates, the corresponding fitted latent quantities for customer $i = 1, \ldots, N$ are obtained via

$$\hat{\theta}_i = \text{sigmoid}(\hat{\eta}_{i1}), \qquad \hat{\eta}_{i1} = \boldsymbol{x}_{1i}'\hat{\boldsymbol{\beta}}_1,$$
$$\hat{\lambda}_i = \exp(\hat{\eta}_{i2}), \qquad \hat{\eta}_{i2} = \boldsymbol{x}_{2i}'\hat{\boldsymbol{\beta}}_2.$$

These quantities should be interpreted as *model-implied estimates* of the latent allocation probability and category intensity, rather than as direct estimates of observable outcomes.

Conditional on an observed response y_i, the latent category size n_i satisfies

$$n_i - y_i \sim \text{Poisson}(\lambda_i(1 - \theta_i)),$$

that is, the unobserved portion of category demand follows a Poisson distribution with mean $\lambda_i(1 - \theta_i)$. Under this conditional distribution, the posterior mean (or optimal predictor under squared-error loss) of the latent category size is given by, see Section 11,

$$E[n_i \mid y_i] = \lambda_i(1 - \theta_i) + y_i.$$

Accordingly, a plug-in estimator of the latent category size for customer i is

$$\hat{n}_i = \hat{\lambda}_i(1 - \hat{\theta}_i) + y_i,$$

which combines the estimated latent intensity and allocation components with the observed focal-brand spending. This estimated quantity summarizes the inferred size of total category demand, while $\hat{\theta}_i$ captures the estimated fraction of that demand allocated to the focal brand.

All continuous covariates are standardized prior to estimation; therefore, coefficients should be interpreted as *relative associations* (approximately per one standard-deviation change for continuous regressors), rather than as marginal effects on observed dollar spending.

Before discussing individual parameter estimates, it is important to clarify the relationship between the covariate set retained in the pogit specification and the broader family of transactional features commonly used in wallet–estimation studies. Following prior work such as [3], a rich collection of transaction–based summaries was initially constructed, including averages, peak values, seasonal aggregates, and concentration ratios over multiple horizons. Many of these variables capture closely related behavioral dimensions and are therefore highly correlated.

8. Estimated Coefficients for the Pogit Model

Table 1 reports the estimated coefficients of the pogit model for the Amazon application, using the full design matrices for both the allocation (SoW) and intensity (SioW) components. The parameters $\hat{\beta}_{1,1}, \ldots, \hat{\beta}_{1,7}$ correspond to the SoW mechanism, while $\hat{\beta}_{2,1}, \ldots, \hat{\beta}_{2,27}$ govern the SioW (latent intensity) mechanism. All confidence intervals

are constructed at the 95% level, and statistically significant p-values at the 5% level are highlighted in blue.

The main sources of heterogeneity in SoW are the Apple–specific RFM covariates computed over the training window T_1. As reported in Table 1, both *Frequency* and *Monetary* display positive and statistically significant associations with the allocation mechanism. In particular, purchase frequency exhibits a strong effect ($\hat{\beta}_{1,3} = 0.622$, p-value < 0.001, $\mathrm{CI}_{95\%} = [0.289, 0.956]$), while cumulative monetary spending also contributes positively and significantly ($\hat{\beta}_{1,4} = 0.347$, p-value $= 0.004$, $\mathrm{CI}_{95\%} = [0.111, 0.582]$). By contrast, *Recency* is not statistically significant in the full specification.

Turning to the SioW (intensity) component, household income and platform usage intensity emerge as key correlates of latent category demand. Among income categories, the *Income 100–149k* group displays a positive and statistically significant association with intensity ($\hat{\beta}_{2,20} = 0.357$, p-value $= 0.021$, $\mathrm{CI}_{95\%} = [0.053, 0.661]$), while the highest income category (*Income* $\geq$*150k*) does not reach conventional significance levels.

Platform engagement, captured by *Use* = *3* (three individuals sharing the Amazon account), exhibits a strong and highly significant positive association with intensity ($\hat{\beta}_{2,25} = 0.589$, p-value < 0.001, $\mathrm{CI}_{95\%} = [0.323, 0.855]$). In addition, one life–event indicator, *Life: Became pregnant*, enters with a statistically significant negative coefficient ($\hat{\beta}_{2,12} = -0.863$, p-value $= 0.027$), suggesting a temporary reduction in latent category demand following this event. Other demographic and life–event controls do not exhibit statistically significant effects at conventional levels.

Table 2 reports the pogit estimates after covariate selection. In the SoW equation, highly redundant transactional summaries are removed, while in the SioW equation a large block of demographic indicators with limited explanatory power is excluded. As a result, the SoW component becomes more parsimonious while preserving its core RFM structure: *Recency*, *Frequency*, and *Monetary* are retained and all three are statistically significant at the 5% level.

Consistent with the full specification, *Frequency* and *Monetary* exhibit positive and precisely estimated effects on the probability of allocating spending to Apple, with estimates $\hat{\beta}_{1,3} = 0.305$ (p-value $= 0.002$, $\mathrm{CI}_{95\%} = [0.114, 0.497]$) and $\hat{\beta}_{1,4} = 0.210$ (p-value $= 0.002$, $\mathrm{CI}_{95\%} = [0.078, 0.341]$), respectively. In contrast to the full model, *Recency* now displays a negative and statistically significant association with SoW ($\hat{\beta}_{1,2} = -0.143$, p-value $= 0.022$, $\mathrm{CI}_{95\%} = [-0.265, -0.020]$), indicating that longer purchase inactivity is associated with a lower propensity to allocate expenditure to Apple.

For the *SioW (intensity)* component, the reduced specification sharpens interpretation by concentrating on household income and platform usage intensity. In particular, the *Income 50–74k* and *Income 100–149k* categories are positively and statistically significantly associated with latent category demand, with estimated coefficients $\hat{\beta}_{2,7} = 0.396$ (p-value $= 0.010$) and $\hat{\beta}_{2,9} = 0.432$ (p-value $= 0.005$), respectively. Platform engagement captured by *Use* = *3* remains strongly and positively associated with intensity ($\hat{\beta}_{2,14} = 0.615$, p-value < 0.001, $\mathrm{CI}_{95\%} = [0.354, 0.875]$). Other retained income and usage categories do not display statistically significant effects at conventional levels.

https://doi.org/10.3390/e1010000

Table 1. Estimated coefficients for the pogit model (full specification). Confidence intervals are at the 95% level. Statistically significant *p*-values (5%) are shown in bold.

Covariate	Estimates	*p*-Value [g,h]	Lower CI	Upper CI
**SoW component**				
Intercept	−0.736	**0.044**	−1.452	−0.021
Recency	−0.155	0.207	−0.396	0.086
Frequency	0.622	<**0.001**	0.289	0.956
Monetary	0.347	**0.004**	0.111	0.582
A.M.Spend [a]	0.158	0.834	−1.320	1.637
M.M.Spend [b]	−0.073	0.919	−1.483	1.337
R.A.M.Units [c]	−0.026	0.866	−0.333	0.280
**SioW/intensity component**				
Intercept	−0.437	0.207	−1.115	0.241
Race: Black [d]	0.345	0.166	−0.144	0.833
Race: White [d]	0.262	0.262	−0.196	0.721
Race: Asian [d]	0.330	0.168	−0.139	0.800
Race: Other [d]	0.234	0.479	−0.414	0.882
Race: Native American [d]	−0.424	0.344	−1.303	0.454
Race: Pacific Islander [d]	−0.606	0.515	−2.431	1.220
Life: Lost job [d]	−0.283	0.093	−0.613	0.047
Life: Moved residence [d]	−0.104	0.379	−0.335	0.128
Life: Divorce [d]	0.132	0.792	−0.849	1.113
Life: Had a child [d]	0.003	0.991	−0.523	0.529
Life: Became pregnant [d]	−0.863	**0.027**	−1.630	−0.096
Age 25–34 [e]	0.027	0.851	−0.255	0.309
Age 35–44 [e]	−0.032	0.843	−0.345	0.281
Age 45–54 [e]	0.169	0.309	−0.156	0.493
Age 55–64 [e]	0.263	0.183	−0.124	0.649
Age $\geq$ 65 [e]	−0.499	0.153	−1.183	0.186
Income 50–74 k [e]	0.326	**0.035**	0.022	0.630
Income 75–99 k [e]	0.097	0.572	−0.239	0.433
Income 100–149 k [e]	0.357	**0.021**	0.053	0.661
Income $\geq$ 150 k [e]	0.301	0.070	−0.025	0.626
Income $<$ 25 k [e]	−0.016	0.941	−0.438	0.406
Income: Prefer not to say [e]	−0.021	0.963	−0.909	0.867
Use = 2 [e]	−0.023	0.840	−0.247	0.201
Use = 3 [e]	0.589	<**0.001**	0.323	0.855
Use = 4+ [e]	0.281	0.131	−0.084	0.646
States [f]	0.014	0.748	−0.069	0.096

[a] A.M.Spend: average monthly spend (USD) over the last 12 months. [b] M.M.Spend: maximum monthly spend (USD) over the last 12 months. [c] R.A.M.Units: ratio of average monthly units to peak monthly units (12-month window). [d] Race, Life: multiple-selection indicator variables. [e] Age, Income, Use: single-selection factor indicators. [f] States: number of distinct shipping states. [g] The *p*-values were computed assuming normality. [h] The underlying variances were computed with the open product gradient method.

Table 2. Estimated coefficients for the pogit model after covariate selection. Confidence intervals are at the 95% level. Statistically significant p-values (5%) are shown in bold.

Covariate	Estimates	p-Value [c,d]	Lower CI	Upper CI
**SoW component**				
Intercept	−2.390	0.123	−5.429	0.650
Recency	−0.143	**0.022**	−0.265	−0.020
Frequency	0.305	**0.002**	0.114	0.497
Monetary	0.210	**0.002**	0.078	0.341
**SioW/intensity component**				
Intercept	1.078	0.445	−1.691	3.848
Life: Lost job [a]	−0.122	0.438	−0.431	0.187
Life: Moved residence [a]	−0.136	0.238	−0.362	0.090
Life: Divorce [a]	0.088	0.860	−0.885	1.060
Life: Had a child [a]	0.024	0.925	−0.483	0.531
Life: Became pregnant [a]	−0.858	**0.026**	−1.610	−0.105
Income 50–74 k [b]	0.396	**0.010**	0.093	0.700
Income 75–99 k [b]	0.236	0.160	−0.093	0.565
Income 100–149 k [b]	0.432	**0.005**	0.132	0.733
Income $\geq$ 150 k [b]	0.318	0.059	−0.012	0.648
Income $<$ 25 k [b]	0.068	0.750	−0.350	0.486
Income: Prefer not to say [b]	0.170	0.693	−0.674	1.014
Use = 2 [b]	0.032	0.776	−0.186	0.250
Use = 3 [b]	0.615	$<$**0.001**	0.354	0.875
Use = 4+ [b]	0.277	0.126	−0.078	0.633

a Life: multiple-selection indicator variables. [b] Income, Use: single-selection factor indicators. [c] The p-values were computed assuming normality. [d] The underlying variances were computed with the open product gradient method.

Overall, the comparison between the full and reduced specifications indicates that the core determinants of both allocation (SoW) and intensity (SioW) remain stable across model stages. This robustness supports the interpretation of the pogit framework as capturing two distinct and persistent latent mechanisms: brand–specific allocation behavior and overall category demand intensity under partial observability.

9. Measures of Performance

To evaluate predictive performance, we compare pogit-based estimates to empirical, Amazon-bounded benchmarks constructed over the evaluation window T_2. Let $\widehat{\theta}_i$ and $\widehat{n}_i$ denote, respectively, the estimated SoW and SioW for customer i obtained from the fitted pogit model. Let θ_i^{Amz} denote the empirical Amazon-bounded SoW benchmark in T_2, computed as Apple spending divided by total technology/electronics spending on Amazon, and let n_i^{emp} denote the corresponding *empirical proxy* for SioW on the model's discretized scale, obtained by dividing total Amazon technology/electronics spending in T_2 by the discretization constant c (estimated using training data only) and applying the same rounding rule used for y_i.

We use the same discretization constant c to express both y_i and n_i^{emp} on a *common normalized (count-like) scale*, so that the empirical Amazon-bounded category volume proxy is comparable to the latent exposure scale implied by the pogit model.

Performance is assessed on the held-out test set $\mathcal{I}_{\mathrm{te}}$, with cardinality $N_{\mathrm{te}} = |\mathcal{I}_{\mathrm{te}}|$, using the Mean Absolute Error (MAE). For SoW, the MAE is defined as

$$\mathrm{MAE}_{\mathrm{SoW}} = \frac{1}{N_{\mathrm{te}}} \sum_{i \in \mathcal{I}_{\mathrm{te}}} \left| \widehat{\theta}_i - \theta_i^{\mathrm{Amz}} \right|,$$

while for SioW, MAE is computed on the discretized (count-like) scale as

$$\mathrm{MAE}_{\mathrm{SioW}} = \frac{1}{N_{\mathrm{te}}} \sum_{i \in \mathcal{I}_{\mathrm{te}}} \left| \widehat{n}_i - n_i^{\mathrm{emp}} \right|.$$

These metrics quantify the average absolute deviation between pogit-based estimates and *Amazon-bounded proxy benchmarks* constructed from observed Amazon transactions in T_2. Because the pogit model targets the *cross-channel* quantities θ_i and n_i (defined over all retail channels), whereas θ_i^{Amz} and n_i^{emp} are computed from *Amazon-only* spending, the resulting MAE values should be interpreted as *proxy-based validation* rather than as market-wide prediction error.

It is important to note that the empirical benchmark n_i^{emp} is constructed from raw Amazon spending in T_2 and is not winsorized. As a result, the upper tail of n_i^{emp} may contain extreme realizations that are intentionally not targeted by the pogit intensity specification under partial observability. Consequently, $\mathrm{MAE}_{\mathrm{SioW}}$ should be interpreted as a proxy-based discrepancy measure that is inherently sensitive to tail behavior, rather than as a measure of pointwise prediction accuracy for extreme outcomes.

Estimating customer wallet quantities under partial observability is intrinsically challenging. In the present application, the Mean Absolute Error (MAE) for SoW equals 0.320 when using the full set of covariates and 0.159 after covariate selection (Table 3). Turning to the intensity dimension, the MAE for SioW equals 5.400 under the full specification and 5.140 after covariate selection. Relative to the empirical support observed in the test set—approximately $[0, 83]$ on the discretized latent scale—this corresponds to about 6% of the observed maximum.

In particular, the SoW MAE reflects the extent to which the inferred allocation signal aligns with a feasible proxy defined on a bounded scale, indicating recovery of a stable and economically interpretable allocation pattern. For the intensity component, the SioW MAE reflects average deviations with respect to an empirical proxy that is intentionally not winsorized and exhibits a heavy upper tail.

Table 3. Mean Absolute Error (MAE) for SoW and SioW estimates using the pogit model. The second column reports results obtained with the full set of covariates $(\boldsymbol{x}_1, \boldsymbol{x}_2)$, while the third column reports results after covariate selection.

Metric	MAE (full $\boldsymbol{x}_1, \boldsymbol{x}_2$)	MAE (after selection)
SoW	0.320	0.159
SioW	5.400	5.140

The observed MAE is consistent with the intended role of SioW as a latent, regularized proxy for overall category demand under partial observability, rather than as a pointwise predictor of extreme outcomes. Three aspects to consider are the following:

First, the pogit model is estimated on a discretized latent scale. Empirical category spending in the evaluation window T_2 is rescaled by a constant c, estimated exclusively on the training set, and rounded to obtain a count–like proxy for latent demand intensity. This transformation implicitly regularizes extreme spending values and ensures that both observed responses and latent intensities are defined on a common, normalized scale.

Second, data–adaptive preprocessing steps—including the removal of extreme outliers using fixed, application–specific rules and the subsequent winsorization of covariates—are learned solely on the training set and applied unchanged to the test set. While this procedure preserves the relative ordering of typical observations, it deliberately limits the influence of rare and extreme realizations that are weakly predictable from observed covariates under partial observability.

Third, the log–linear specification of the intensity component, $\lambda_i = \exp(\boldsymbol{x}'_{2i}\boldsymbol{\beta}_2)$, combined with standardized covariates and moderate coefficient magnitudes, implies that very large estimated SioW values would require multiple covariates to simultaneously attain extreme values. Such configurations are intentionally rare after preprocessing and are therefore not extrapolated by the model.

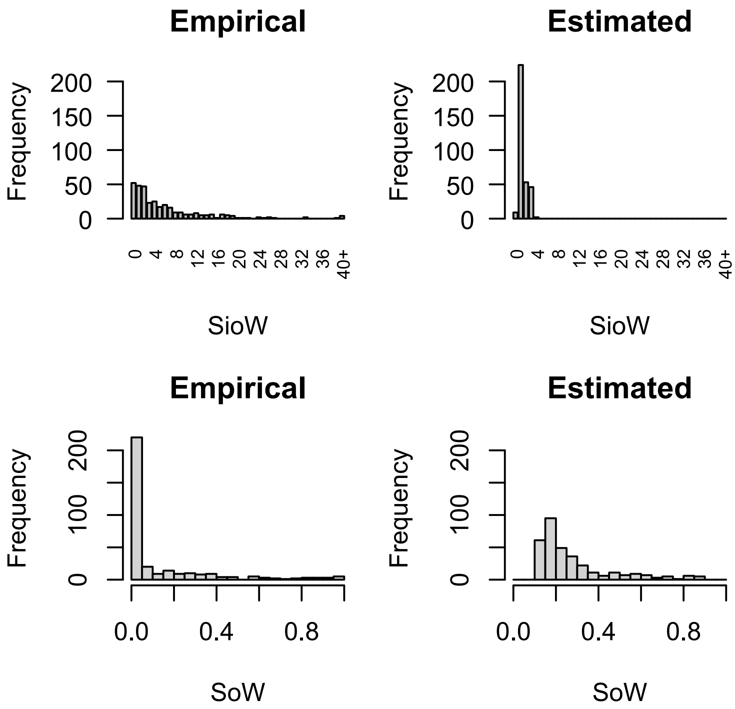

Figure 1. Complete design matrices: Empirical Amazon–bounded benchmarks and pogit–implied estimates on the test set for Size–of–Wallet (SioW, top row) and Share–of–Wallet (SoW, bottom row). Empirical distributions are constructed from observed Amazon spending during the evaluation window and discretized to match the latent modeling scale, while estimated distributions are obtained from the fitted pogit model under partial observability.

To complement the scalar MAE summaries in Table 3, Figures 1 and 2 provide a distributional comparison between empirical Amazon–bounded benchmarks and pogit–implied estimates on the test set. These figures illustrate how the aggregate MAE values arise from systematic differences in distributional shape, particularly in the upper tail of the SioW component.

Together, these mechanisms clarify that the pogit model prioritizes the recovery of stable and economically interpretable latent demand patterns under partial observability, rather than extrapolation toward extreme realizations that are weakly identifiable from the available covariates.

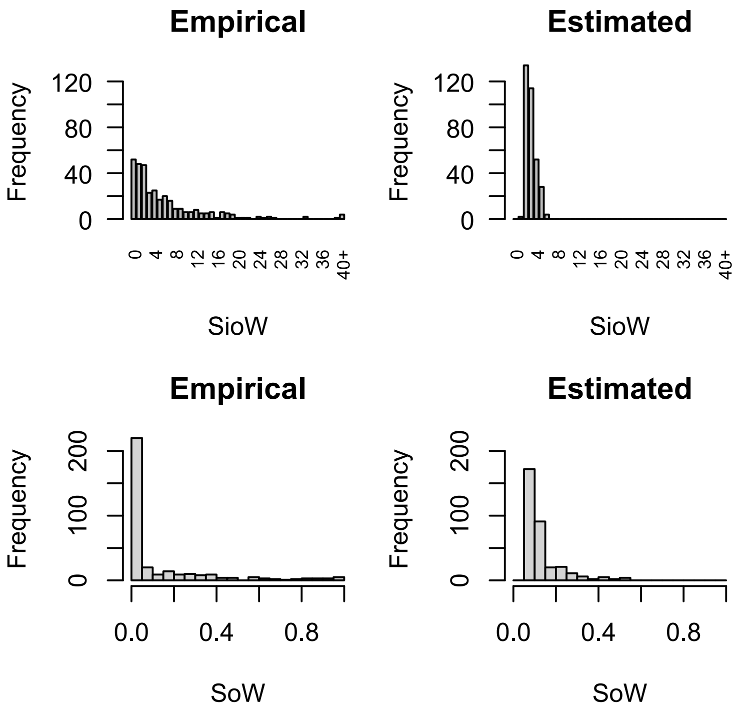

Figure 2. Design matrices with variable selection: Empirical Amazon–bounded benchmarks and pogit–implied estimates on the test set for Size–of–Wallet (SioW, top row) and Share–of–Wallet (SoW, bottom row). Empirical distributions are constructed from observed Amazon spending during the evaluation window and discretized to match the latent modeling scale, while estimated distributions are obtained from the fitted pogit model under partial observability.

10. Catalog of Predictor Variables

10.1. Catalog of Predictor Variables for SoW Modeling

1. `race_black_or_african_american`: Indicator (=1) if the respondent selected *Black or African American*.
2. `race_asian`: Indicator (=1) if the respondent selected *Asian*.
3. `race_other`: Indicator (=1) if the respondent selected *Other*.
4. `race_american_indian_native_american_or_alaska_native`: Indicator (=1) if the respondent selected *American Indian/Native American or Alaska Native*.
5. `race_native_hawaiian_or_other_pacific_islander`: Indicator (=1) if the respondent selected *Native Hawaiian or Other Pacific Islander*.
6. `life_lost_a_job`: Indicator (=1) *Lost a job*.
7. `life_moved_place_of_residence`: Indicator (=1) *Moved place of residence*.
8. `life_had_a_child`: Indicator (=1) *Had a child*.
9. `life_became_pregnant`: Indicator (=1) *Became pregnant*.
10. `life_n_selected`: Count of selected life changes.
11. `life_is_multiple`: Indicator (=1) if more than one life change was selected.
12. `q_demos_age_25 - 34 years`: Indicator (=1) age 25–34.
13. `q_demos_age_35 - 44 years`: Indicator (=1) age 35–44.
14. `q_demos_age_45 - 54 years`: Indicator (=1) age 45–54.
15. `q_demos_age_55 - 64 years`: Indicator (=1) age 55–64.
16. `q_demos_age_65 and older`: Indicator (=1) age 65+.

17. q_demos_hispanic_Yes: Indicator (=1) Hispanic/Latino origin.
18. q_demos_education_Graduate or professional degree (MA, MS, MBA, PhD, JD, MD, DDS, etc): Indicator (=1) graduate/professional degree.
19. q_demos_education_High school diploma or GED: Indicator (=1) high school diploma or GED.
20. q_demos_education_Prefer not to say: Indicator (=1) prefer not to say (education).
21. q_demos_education_Some high school or less: Indicator (=1) some high school or less.
22. q_demos_income_$50,000 - $74,999: Indicator (=1) income USD 50,000–74,999.
23. q_demos_income_$75,000 - $99,999: Indicator (=1) income USD 75,000–99,999.
24. q_demos_income_$100,000 - $149,999: Indicator (=1) income USD 100,000–149,999.
25. q_demos_income_$150,000 or more: Indicator (=1) income USD 150,000 or more.
26. q_demos_income_Less than $25,000: Indicator (=1) income less than USD 25,000.
27. q_demos_income_Prefer not to say: Indicator (=1) prefer not to say (income).
28. q_demos_gender_Male: Indicator (=1) male.
29. q_demos_gender_Other: Indicator (=1) *Other* gender identity.
30. q_demos_gender_Prefer not to say: Indicator (=1) prefer not to say (gender).
31. q_sexual_orientation_LGBTQ+: Indicator (=1) identifies as LGBTQ+.
32. q_sexual_orientation_prefer not to say: Indicator (=1) prefer not to say (sexual orientation).
33. q_substance_use_1_No: Indicator (=1) no cigarette use in household/account group.
34. q_substance_use_1_Prefer not to say: Indicator (=1) prefer not to say (cigarettes).
35. q_substance_use_1_Yes: Indicator (=1) cigarette use present.
36. q_substance_use_2_No: Indicator (=1) no marijuana use in household/account group.
37. q_substance_use_2_Prefer not to say: Indicator (=1) prefer not to say (marijuana).
38. q_substance_use_2_Yes: Indicator (=1) marijuana use present.
39. q_substance_use_3_No: Indicator (=1) no alcohol use in household/account group.
40. q_substance_use_3_Prefer not to say: Indicator (=1) prefer not to say (alcohol).
41. q_substance_use_3_Yes: Indicator (=1) alcohol use present.
42. q_personal_1_Prefer not to say: Indicator (=1) prefer not to say (diabetes).
43. q_personal_1_Yes: Indicator (=1) diabetes present.
44. q_personal_2_Prefer not to say: Indicator (=1) prefer not to say (wheelchair use).
45. q_personal_2_Yes: Indicator (=1) wheelchair use present.
46. q_amazon_use_howmany_2: Indicator (=1) two people share the Amazon account.
47. q_amazon_use_howmany_3: Indicator (=1) three people share the account.
48. q_amazon_use_howmany_4+: Indicator (=1) four or more people share the account.
49. q_amazon_use_hh_size_2: Indicator (=1) household size = 2.
50. q_amazon_use_hh_size_3: Indicator (=1) household size = 3.
51. q_amazon_use_hh_size_4+: Indicator (=1) household size ≥ 4.
52. n_states: Number of distinct shipping states (excludes placeholder values such as digital/locker).
53. dollar_winter_avg_12m: Average monthly spend (USD) in Winter over the last 12 months.
54. products_winter_avg_12m: Average monthly units in Winter over the last 12 months.
55. dollar_spring_avg_12m: Average monthly spend (USD) in Spring (Mar–Apr–May) over the last 12 months.
56. products_spring_avg_12m: Average monthly units in Spring over the last 12 months.

https://doi.org/10.3390/e1010000

57. dollar_autumn_avg_12m: Average monthly spend (USD) in Autumn (Sep–Oct–Nov) over the last 12 months.
58. products_autumn_avg_12m: Average monthly units in Autumn over the last 12 months.
59. dollar_summer_avg_12m: Average monthly spend (USD) in Summer (Jun–Jul–Aug) over the last 12 months.
60. products_summer_avg_12m: Average monthly units in Summer over the last 12 months.
61. dollar_avg_12m: Average monthly spend (USD) over the last 12 months (months with zero spend included).
62. products_avg_12m: Average monthly units over the last 12 months (months with zero units included).
63. dollar_std_12m: Standard deviation of monthly spend over the last 12 months (volatility).
64. products_std_12m: Standard deviation of monthly units over the last 12 months.
65. dollar_top1_12m: Maximum monthly spend (USD) within the last 12 months.
66. products_top1_12m: Maximum monthly units within the last 12 months.
67. dollar_top3_avg_12m: Average spend (USD) across the top three months (last 12 months).
68. products_top3_avg_12m: Average units across the top three months (last 12 months).
69. dollar_top6_avg_12m: Average spend (USD) across the top six months (last 12 months).
70. products_top6_avg_12m: Average units across the top six months (last 12 months).
71. dollar_avg12_top1_ratio: Ratio of the 12-month average monthly spend to the peak month's spend.
72. products_avg12_top1_ratio: Ratio of the 12-month average monthly units to the peak month's units.
73. dollar_avg12_top3_ratio: Ratio of the 12-month average monthly spend to the top-three-months average.
74. products_avg12_top3_ratio: Ratio of the 12-month average monthly units to the top-three-months average.
75. dollar_avg12_top6_ratio: Ratio of the 12-month average monthly spend to the top-six-months average.
76. products_avg12_top6_ratio: Ratio of the 12-month average monthly units to the top-six-months average.
77. months_with_purchase_12m: Number of months (0–12) with at least one purchase (units >0) in the last 12 months.
78. recency_days_2018_Apple: Days from the most recent Apple purchase to ref_date2 (lower = more recent), computed in the RFM history window. Variable names retain the prefix "2018" for legacy reasons; all RFM quantities are computed over T_1 ending on 2021-10-31.
79. frequency_2018_Apple: Count of Apple purchases in the RFM history window (number of orders/rows, or distinct order_id if available).
80. monetary_2018_Apple: Total Apple spend (USD) in the RFM history window.

10.2. Catalog of Response Variables for SoW Modeling and Validation

1. amount_total: Total spending by the customer on technology/electronics products from any brand on Amazon (USD) over T_2.

2. `share_apple_total`: Amazon-bounded Apple share of spend in the technology/electronics universe over T_2, defined as $\text{spend}_i^{\text{Apple}|\text{Amz},\mathcal{U}}/\text{spend}_i^{\text{Amz},\mathcal{U}} \in [0,1]$. This quantity is used only for ex post benchmarking and is not used for estimation.
3. `spend_apple`: Total Apple spending by the customer on Amazon within $\mathcal{U}$ over T_2 (USD), i.e., $\text{spend}_i^{\text{Apple}|\text{Amz},\mathcal{U}}$.
4. `c_scale`: Discretization constant used to construct the count-like response, defined as $c = \widehat{\sigma}_{\text{tr}}^{2\,\text{Apple}|\text{Amz},\mathcal{U}}/\overline{\text{spend}}_{\text{tr}}^{\text{Apple}|\text{Amz},\mathcal{U}}$.
5. `y`: Discretized, count-like proxy for Apple purchase volume on Amazon within $\mathcal{U}$ over T_2, defined as $y_i = \text{round}(\text{spend}_i^{\text{Apple}|\text{Amz},\mathcal{U}}/c)$. It is not a literal count of physical transactions.

11. Estimated SioW

This appendix recalls a standard result for the pogit (Poisson–binomial) model concerning the conditional expectation of the latent category size. The derivation follows directly from the probabilistic structure of the model and has been discussed in related formulations of Poisson–binomial and generalized binomial models (see, e.g., [4]). It is included here solely for completeness and to make the exposition self–contained; no methodological novelty is claimed.

First, observe that $E(n_\ell \mid \boldsymbol{y}) = E(n_\ell \mid y_\ell)$, where

$$
\begin{aligned}
E(n_\ell \mid y_\ell) &= \sum_{n_\ell=0}^{\infty} n_\ell p(n_\ell \mid y_\ell) \\
&= \sum_{n_\ell=0}^{\infty} n_\ell p(y_\ell \mid n_\ell)\frac{p(n_\ell)}{p(y_\ell)} \\
&= \sum_{n_\ell=y_\ell}^{\infty} n_\ell p(y_\ell \mid n_\ell)\frac{p(n_\ell)}{p(y_\ell)} \\
&= \sum_{n_\ell=y_\ell}^{\infty} n_\ell \binom{n_\ell}{y_\ell}\theta_\ell^{y_\ell}(1-\theta_\ell)^{n_\ell-y_\ell}\frac{\frac{e^{-\lambda_\ell}\lambda_\ell^{n_\ell}}{n_\ell!}}{\frac{e^{-\theta_\ell\lambda_\ell}(\theta_\ell\lambda_\ell)^{n_\ell}}{n_\ell!}} \\
&= \sum_{n_\ell=y_\ell}^{\infty} n_\ell(1-\theta_\ell)^{n_\ell-y_\ell}\frac{e^{-(1-\theta_\ell)\lambda_\ell}\lambda^{n_\ell-y_\ell}}{(n_\ell-y_\ell)!}
\end{aligned}
$$

Letting k be defined as $n_\ell - y_\ell$, the following is obtained.

$$
\begin{aligned}
E(n_\ell \mid y_\ell) &= \sum_{k=0}^{\infty}(k+y_\ell)e^{-(1-\theta_\ell)\lambda_\ell}((1-\theta_\ell)\lambda_\ell)^k/k! \\
&= \sum_{k=0}^{\infty}(k+y_\ell)\text{Poisson}(k \mid (1-\theta_\ell)\lambda_\ell) \\
&= \sum_{k=0}^{\infty}k\text{Poisson}(k \mid (1-\theta_\ell)\lambda_\ell) + y_\ell\sum_{k=0}^{\infty}\text{Poisson}(k \mid (1-\theta_\ell)\lambda_\ell) \\
&= (1-\theta_\ell)\lambda_\ell + y_\ell,
\end{aligned}
$$

as claimed.

References

1. Berke, A.; Calacci, D.; Mahari, R.; Yabe, T.; Larson, K.; Pentland, S. Open e-commerce 1.0, five years of crowdsourced U.S. Amazon purchase histories with user demographics. *Sci. Data* **2024**, *11*, 491. https://doi.org/10.1038/s41597-024-03329-6.
2. Glady, N.; Croux, C. Predicting customer wallet without survey data. *J. Serv. Res.* **2009**, *11*, 219–231. https://doi.org/10.1177/1094670508328983.

3. Li, X.; Shemshadi, A.; Olech, Ł.; Michalewicz, Z. Customer wallet share estimation for manufacturers based on transaction data. In *Data Mining*; Springer Singapore: Singapore, 2019; pp. 171–182. https://doi.org/10.1007/978-981-15-1699-3_14.
4. Winkelmann, R.; Zimmermann, K.F. *Poisson-Logistic Regression*; Working Paper 93–18; Department of Economics, University of Munich: Munich, Germany, 1993.

Disclaimer/Publisher's Note: The statements, opinions and data contained in all publications are solely those of the individual author(s) and contributor(s) and not of MDPI and/or the editor(s). MDPI and/or the editor(s) disclaim responsibility for any injury to people or property resulting from any ideas, methods, instructions or products referred to in the content.
